# Emerging nanomaterials for novel wound dressings: From metallic nanoparticles and MXene nanosheets to metal-organic frameworks

**DOI:** 10.1016/j.heliyon.2024.e39611

**Published:** 2024-10-22

**Authors:** Gholamreza Faghani, Amir Azarniya

**Affiliations:** a Department of Mechanical Engineering, Khatam-Ol-Anbia (PBU) University, Tehran, Iran; b Department of Materials Engineering, Tarbiat Modares University, Tehran, Iran

**Keywords:** Antibacterial wound dressings, Wound healing, Metallic nanoparticles, MXenes nanosheets, Metal organic frameworks

## Abstract

The growing need for developing reliable and efficient wound dressings has led to recent progress in designing novel materials and formulations for different kinds of wounds caused by traumas, burns, surgeries, and diabetes. In cases of extreme urgency, accelerating wound recovery is of high importance to prevent persistent infection and biofilm formation. The application of nanotechnology in this domain resulted in the creation of distinct nanoplatforms for highly advanced wound-healing therapeutic approaches. Recently developed nanomaterials have been used as antibacterial agents or drug carriers to control wound infection. In the present review, the authors aim to review the recently published research on the effects of incorporating emerging nanomaterials into novel wound dressings and investigate their distinct roles in the wound healing process. It was determined that the metallic nanoparticles (NPs) exhibit antimicrobial and regenerative properties, metal oxide NPs regulate inflammation and promote tissue regeneration, MXene NPs enhance cell adhesion and proliferation, while metal-organic frameworks (MOFs) offer controlled drug delivery capabilities. Further research is required to fully understand the mechanisms and optimize the applications of these NPs in wound healing.

## Graphical abstract

**Figure:**
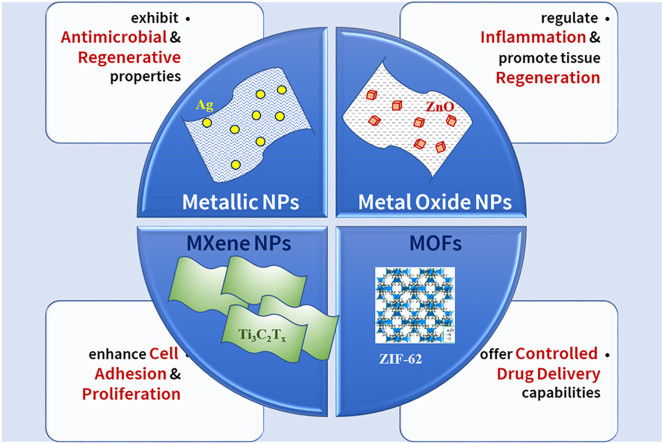

## 1 Introduction

A miscellaneous assortment of bioengineered nanocomposite scaffolds has been recently developed for wound healing, tissue engineering, and sustainable drug delivery. The physicomechanical and biological behavior of the fabricated nano-systems should be able to accommodate the specific requirements of the intended application. More specifically, by carefully selecting nanomaterials with appropriate physical and mechanical properties and ensuring their biocompatibility, one can develop nanocomposite scaffolds that are well-suited for specific wound healing applications [[1], [2], [3]]. Choosing optimized dressings considering several critical wound-related factors including wound type, depth, and amount of exudate is vital for effective course of treatment. An ideal wound dressing can secure and maintain the moist environment necessary for tissue regeneration. Additionally, the selected dressing must hold adequate air permeability and maintain the moisture while removing exudate from the wound’s surface. They should also be able to provide an antimicrobial medium acting as a barrier against pathogenic microorganisms. The bandage needs to be affordable, biocompatible, easy to apply, and require less changing [[4], [5], [6], [7], [8], [9], [10], [11], [12]].

Nanotechnology offers novel approaches to regenerative medicine, including the use of metallic NPs (MNPs) and MOFs. Various biocompatible NPs have recently been suggested for improved wound healing [13,14]. MNPs such as silver, gold, and zinc oxide have shown beneficial features including low in vivo toxicity as well as bacteriostatic and bactericidal activity. NPs demonstrate improved physicochemical characteristics due to their smaller mean size, increased surface area and aspect ratio. They can affect antibacterial, anti-inflammatory, and angiogenic activity of the wound microenvironment by stimulating various cellular and molecular processes, and changing it from a non-healing to a healing environment [[15], [16], [17], [18]].

Because of concerns about antibiotic resistance of novel therapeutic approaches such as MNPs, more research have been recently conducted on different aspects of wound healing applications of these approaches [19]. Various MNPs such as silver [20,21], gold [22], copper [[23], [24], [25]], titanium [26], zinc oxide [27], iron oxide [28], magnesium oxide [29], and their derivatives are known to have antibacterial effects and are widely used in the design of novel wound dressings. According to the literature, MNPs such as Ag, Au, and CuNPs, have demonstrated great promise in wound healing due to their distinct physicochemical characteristics. AgNPs display prominent antimicrobial activity, which can in turn prevent infection and improve the wound healing process [30]. AuNPs possess anti-inflammatory features enabling them to enhance tissue regeneration [31]. CuNPs have been shown to improve wound closure and stimulate angiogenesis [32]. Metal oxide NPs, including ZnO and TiO_2_NPs, have also demonstrated significant wound healing qualities. ZnONPs and TiO_2_NPs exhibit antimicrobial properties and while ZnONPs can regulate inflammation [33], TiO_2_NPs are able to promote tissue regeneration [34]. MXene nanomaterials possess excellent mechanical strength, electrical conductivity, and biocompatibility. These properties allow them to be used as scaffolds and wound dressings, promoting cell adhesion, proliferation, and tissue regeneration [35]. MOFs’ distinct porous structure and adjustable properties have drawn attention in recent years. MOFs can be used in wound healing applications as controlled drug delivery systems. They can encapsulate therapeutic agents, providing sustained release and targeted delivery to the wound site [36].

In this paper, we first aimed to briefly review the wound healing mechanism, antibacterial behavior, angiogenesis and immunomodulation processes. The nanotechnology-driven advantages as well as recent studies based on the novel compositions and formulations for developing efficient wound dressings, including MNPs, MXenes, and MOFs, and the relevant healing mechanisms are briefly outlined. Finally, the clinical trials carried out to evaluate the therapeutic capabilities of the nanoparticles are summarized based on the data published in FDA-supported ClinicalTrials.gov databank and, accordingly, the future research perspectives are outlined.

## 2 Biomedical applications of nanomaterials

Nanomaterials exhibit exceptional physical and chemical properties compared to their bulk counterparts, including enhanced conductivity, catalytic activity, and antimicrobial characteristics. These unique attributes have prompted their exploration across various industries, notably in biomedical applications [37]. This section outlines the specific utilization of nanomaterials within this domain, focusing on their role in drug delivery, photoablation therapies, biosensor development, and wound dressing. Table 1 provides specific examples and summarizes some recent advancements in each of these areas to demonstrate the potential of nanomaterials in addressing critical healthcare challenges.

**Table 1:** Biomedical applications and nanotechnology-driven advantages of different types of nanomaterials.

Nanomaterial	Microstructure/properties	Biomedical application	Nanotechnology-driven advantage	Ref.
**Core-shell MOF nanostructures**	Core: Inorganic NPs including magnetic, quantum dots (QDs), Au, and gadolinium NPs Shell: MOF	Targeted drug delivery; Imaging-guided cancer therapy	High drug loading capacity; Controlled drug release (sustained and stimuli-responsive); Enhanced imaging capabilities (multimodal imaging); Improved biocompatibility and biodegradability; Potential for synergistic therapeutic effects	[38]
**AuNR-rGO@PCL nanofibrous scaffold**	Higher mechanical strength (7-fold increase); Increased electrical conductivity (6 times); Larger surface area (6.4 times); Enhanced NIR light absorbance	Photoablation treatment (Breast cancer phototherapy); Nerve regeneration	Efficient breast cancer cell ablation under low laser power density; Enhanced nerve cell growth and differentiation; Potential for dual functionality as a biomaterial; Improved physicochemical properties leading to enhanced performance	[39]
**Biotin/PEG-UCNPs (Upconversion NPs)**	UCNPs composition: KMnF_3_:18%Yb, 2%Er; High longitudinal relaxivity (6.124 mM^−1^ s^−1^) for MRI	Glioblastoma imaging & surgery guidance	Improved tumor margin delineation; Enhanced contrast effect in MRI; Targeted delivery to glioblastoma cells via biotin ligand; Negligible in vivo toxicity; Potential for more accurate surgical resection	[40]
**CdS-CuInS**_**2**_**-CQDs (carbon QDs) heterojunction**	Efficient energy level matching between CdS and CuInS_2_; Upconversion luminescence properties of CQDs; Enhanced light absorption and photocurrent generation	Biosensing; Detection of prostate-specific antigen (PSA) for prostate cancer diagnosis	High sensitivity and selectivity for PSA detection; Improved optical performance and signal amplification; Enhanced photocurrent generation; Potential for application in other disease biomarker detection	[41]
**CeO**_**2**_**NPs-encapsulated chitosan NPs electrosprayed on electrospun PCL/cellulose acetate-based nanofibrous membrane**	Uniform distribution of cerium oxide (CeO_2_NPs)-chitosan NPs within the nanofibrous matrix	Diabetic wound healing	Enhanced antioxidant activity; Improved cell viability and migration; Significant reduction in bacterial growth; Accelerated diabetic wound healing; Biocompatibility and non-toxicity	[42]

### 2.1 Targeted drug delivery

Conventional chemotherapy relies on the circulatory system to distribute anticancer drugs throughout the body, often resulting in systemic toxicity due to indiscriminate targeting of both healthy and cancerous cells. To address this challenge, targeted drug delivery systems have emerged as a promising therapeutic strategy [37]. By encapsulating therapeutic agents within nanocarriers, these systems aim to enhance drug concentration at the tumor site while minimizing off-target effects [43]. Magnetic nanoparticles have shown particular promise as drug carriers due to their ability to be manipulated by external magnetic fields. To achieve targeted delivery, these nanoparticles are frequently coated with biocompatible materials and loaded with anticancer drugs. Upon administration, an external magnetic field is applied to guide the drug-loaded nanoparticles to the desired tumor location [44].

MOFs have emerged as a promising platform for targeted drug delivery due to their unique structural and chemical properties. Their ability to encapsulate drugs, facilitate controlled release, and be modified with targeting ligands has driven significant research efforts. While MOFs offer advantages in passive, active, and stimulus-responsive targeting strategies, challenges such as elucidating specific targeting mechanisms, translating laboratory findings to clinical settings, and achieving large-scale production persist. To realize the full potential of MOFs in cancer therapy, future research should focus on understanding fundamental targeting mechanisms, collaborating with pharmaceutical industries for clinical translation, and developing scalable synthesis methods. The application of MOFs in tumor-targeted drug delivery systems has been comprehensively examined by Kong et al. [45].

### 2.2 Photoablation treatment

Photodynamic therapy (PDT) and photothermal therapy (PTT) are two photoablation techniques utilizing light to induce cell death. PDT employs photosensitizers to generate ROS upon light irradiation, leading to cellular damage. PTT, on the other hand, converts light energy into heat, causing thermal ablation of cancerous cells. For instance, TiO_2_NPs exhibit promising properties for both modalities due to their photocatalytic activity. Upon light irradiation, TiO_2_ generates ROS through electron-hole pair formation and subsequent reactions with water and oxygen, resulting in cell death. Additionally, it can convert light into heat, enabling PTT. These combined effects make TiO_2_NPs a promising candidate for cancer therapy [46,47].

### 2.3 Bioimaging

Bioimaging, a non-invasive technique, enables real-time visualization of biological processes in living cells/organisms. NPs, particularly magnetic NPs, have emerged as versatile tools for bioimaging due to their customizable properties and ability to overcome limitations of traditional contrast agents. Their unique optical, magnetic, and structural characteristics enable applications in various imaging modalities. While challenges such as toxicity, biodistribution, and image quality persist, ongoing research is focused on developing NPs with improved performance and clinical translation potential. NPs properties, including size, shape, surface chemistry, and charge, significantly influence cellular uptake and intracellular trafficking. Surface modifications with ligands or biomolecules can enhance specific targeting and cellular internalization, while careful consideration of nanoparticle dimensions is crucial for optimal biodistribution and endosomal escape [48].

### 2.4 Biosensing

Biosensors, comprising bioreceptors, transducers, and signal processors, enable the detection and quantification of biological analytes. These devices find applications across various industries, including environmental monitoring, pharmaceuticals, food safety, and healthcare. Transducer types, such as electrochemical, optical, and piezoelectric, determine the specific analytical approach. By converting biological interactions into measurable electrical signals, biosensors provide valuable insights into biological systems and processes [49]. For instance, MXenes, a class of 2D nanomaterials, offer unique properties for biosensing and other biomedical applications. Their high sensitivity, tunable properties, and biocompatibility make them promising candidates for various diagnostic and therapeutic applications. However, challenges related to synthesis, biocompatibility, and long-term stability need to be addressed to realize their full potential. Future research should focus on developing scalable synthesis methods, improving biocompatibility, and exploring novel applications of MXenes in biosensing and nanomedicine [50].

## 3 Mechanisms of action of nanomaterials in wound healing

### 3.1 Wound healing mechanism

Hemostasis, inflammation, granulation or proliferation, and maturation with scar tissue formation are considered as the main successive stages of the human wound-healing process [51,52]. The following events indicate the major stages of healing process which could be respectively identified as (i) vascular construction, aggregation of the platelets, degranulation, and fibrin formation (thrombus); (ii) neutrophil infiltration, monocyte infiltration followed by differentiation to macrophage, and lymphocyte infiltration; (iii) re-epithelialization, angiogenesis, synthesis of collagen, and extracellular matrix (ECM) formation; and (iv) collagen remodeling, vascular maturation and regression [53]. Successful healing of a wound requires the sequential implementation of all the above-mentioned steps in their specific time frames. Any deviations from these steps will cause a delay in the wound restoration process, leading to the development of chronic wounds [54,55].

**Fig. 1:**
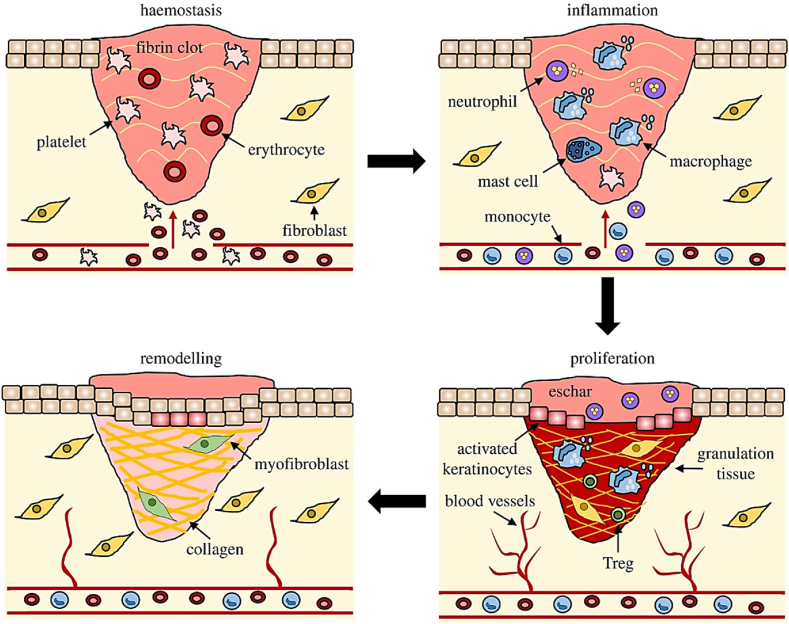
Schematic illustration of four different stages of wound healing process including hemostasis, inflammation, granulation, and maturation with scar tissue formation [57] (Published by the Royal Society under the terms of the Creative Commons Attribution License http://creativecommons.org/licenses/by/4.0/).

The removal of debris and bacteria, coagulation of exudates, and blood clotting along with the simultaneous activation of reactive oxygen species (ROS), proteases, growth factors, and cytokines are the key stages of hemostasis and inflammation. Afterwards, formation of granulation tissue, angiogenesis, and EMC secretion take place with the beginning of the proliferation phase and subsequent migration of epithelial cells. Eventually, scar tissue is formed as a result of wound contraction and reepithelization in the maturation stage [53,56]. The quadruple stages of the wound repair process along with their relevant cellular and bio-physiologic events are schematically illustrated in Fig. 1.

Wound healing process is mediated by various molecular pathways and different cell types involved including (i) neutrophils, macrophages, and T-lymphocytes, (ii) fibroblasts, (iii) endothelial, and (iv) stem cells. Neutrophils contribute to the removal of bacteria, necrotic material, and cell debris by releasing ROS and proteases in the wound environment [53]. On the other hand, macrophages participate in wound healing by producing cytokines, inducing and clearing apoptotic cells, and promoting the proliferation phase after the phenotypic transition to reparative mode [[58], [59], [60]]. It was suggested that efferocytosis, the clearance of apoptotic cells by phagocytes, is crucial for the maintenance of tissue homeostasis and prevention of inflammation [61,62]. Furthermore, T-lymphocytes are activated in the inflammation and re-epithelialization phases [63,64] and facilitate the healing process using the epidermal growth factor receptor pathway [65]. Endothelial cells play an important role in hemostasis phase and formation and growth of new tissue [66]. Fibroblasts support collagen production and granulation tissue formation, and produce ECM components including glycosaminoglycans and proteoglycans. In addition, myofibroblasts are thought to mediate physical contraction of wounds [53,67,68]. Finally, stem cells promote wound healing process by releasing growth factors, inducing paracrine signaling and stimulating regeneration of damaged tissue [69]. Mesenchymal stem cells differentiate into fibroblasts, osteoblasts, keratinocytes, chondrocytes, and adipocytes. Keratinocytes, for instance, arise from the epidermal stem cells and then re-epithelialize the wound [53].

### 3.2 Antibacterial behavior

**Fig. 2:**
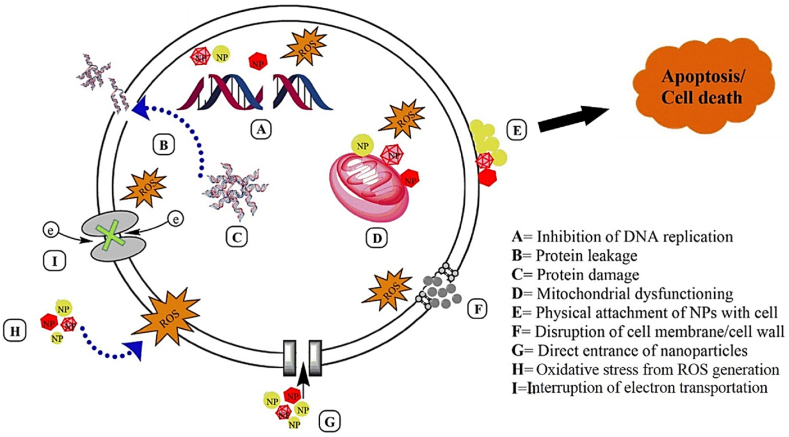
Schematic illustration of potential antibacterial mechanisms of action of MNPs (Reprinted with permission from Ref. [71] Copyright 2019 Springer Link).

Various nanoparticles exhibit strong antibacterial activity with possible mechanisms of bacterial killing and triggering different interconnected pathways including (i) excessive release of ROS inside microbes, (ii) production and accumulation of metal ions in microbial membranes, (iii) causing biomolecular damage, (iv) disruption of metabolic activities via electrostatic attraction between metal nanoparticles and microbial cells, (v) damage in microbial plasma membranes which disrupt vital respiratory chain enzymes, (vi) increase in H_2_O_2_ generation by which microbial proteins/enzymes are inhibited, (vii) ATP depletion, and (viii) particle-membrane interaction. Moreover, they potentially suppress the evolution of more resistant bacteria via simultaneous targeting of multiple biomolecules and subsequent prevention of resistant strains development [[70], [71], [72]]. The potential antibacterial mechanisms of metal nanoparticles are schematically illustrated in Fig. 2.

### 3.3 Angiogenesis

**Fig. 3:**
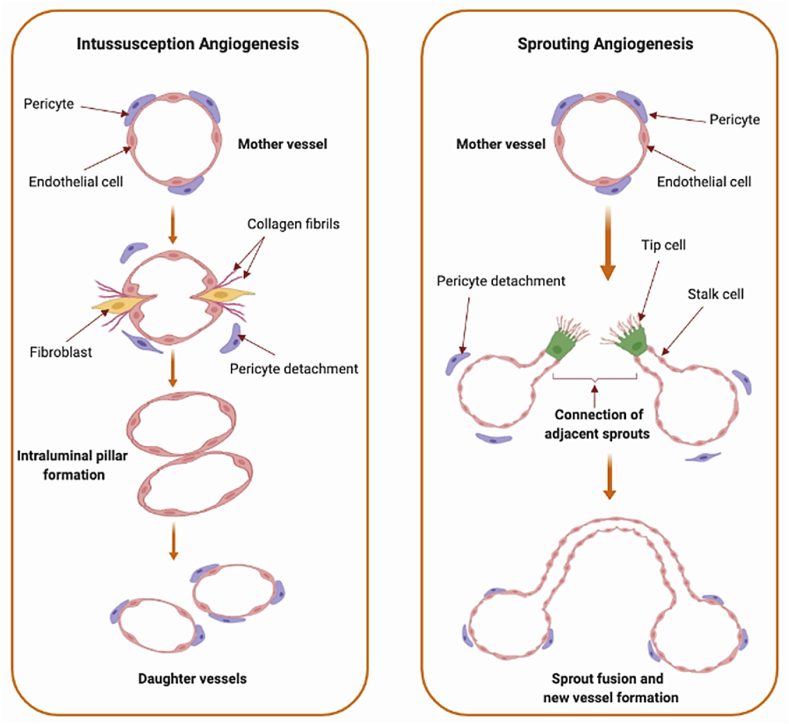
Schematic illustration of angiogenic growth mechanisms including intussusceptive (left) and sprouting angiogenesis (right) (Reprinted with permission from Ref. [73]. Copyright 2023 Taylor & Francis Online).

During angiogenesis, preexistent vessels develop and form new blood vessels. In this highly regulated process, pro- and anti-angiogenic factors interact to mediate the transition of vascular endothelium through various stages of vascular growth. Angiogenic vascular development involves multiple successive stages including activation, progression, migration, differentiation, and maturation of endothelial cells. During the activation phase, vascular permeability increases and pericytes detach away from endothelial cells. During the progression phase, endothelial cells destabilize and migrate as a result of the degradation of extracellular matrix (ECM). This process is regularly mediated by chymases, heparanases, angiopoietins, and matrix metalloproteinases, which work together to promote the degradation of matrix molecules and facilitate the formation of new blood vessels by activated endothelial cells. At the final stages of angiogenesis, pericyte attachment and the resumption of the quiescent endothelial cells lead to the maturation and stabilization of the new vessels [[73], [74], [75]]. Two major angiogenesis mechanisms, known as sprouting and intussusception are schematically shown in Fig. 3.

### 3.4 Immunomodulation

Organizing the wound repair process is highly mediated by the immune system. Therefore, a strategic approach to accelerated wound healing is to control the immune system through coordinating immune cells activities for producing diverse inflammatory and anti-inflammatory cytokines known as immunomodulation. Various biomaterials, bioactive anti-inflammatory agents, antigens, and immunomodulators, and cell delivery have been utilized to optimize the process [76,77]. Multiple immunomodulatory approaches to promote wound healing process are described as follows; (i) pharmacological agents such as non-steroidal anti-inflammatory drugs (NSAIDs) [78], COX-2 Inhibitor (Celecoxib) [79], inducible nitric oxide synthase (iNOS) inhibitor (1400W) [80], Manganese superoxide dismutase (MnSOD) mimetic molecule [81], and injectable Zn-Al layer double hydroxide nanocomposites loaded with curcumin [82], (ii) biological and synthetic approaches such as adjusting surface topography and hydrophilic features [83], heparin-immobilized copolymers of L-lactide (LA) and 5-methyl-5-benzyloxycar-bonate-1,3-dioxan-2-one (MBC) on metal stents [84], surgical mesh materials developed by biological amendments [85], dermal ECM (D-ECM) or urinary bladder matrix ECM (UBM-ECM) coating polypropylene mesh [86], keratin and collagen films [87], (iii) cell and cytokine therapies including macrophage polarization [87], mesenchymal stem cells (MSCs) [88], and human bone marrow stromal cells (BM-SC) [89], and (iv) utilizing cell secretome and extracellular vesicles such as MSCs extracellular (EVs) [90] and amniotic fluid stem cell-derived extracellular vesicle [91].

## 4 Metallic nanoparticles for wound healing

### 4.1 Ag nanoparticles

Silver NPs (AgNPs) are the most studied, highly utilized, and commercialized nanomaterial for skin regeneration with anti-bactericidal activity and potential to stimulate skin tissue regeneration. These features enable them to effectively prevent wound infections and accelerate the healing process of injured tissue compared to conventional topical treatments [20]. Being effective against a wide range of microorganisms, AgNPs can exert their effects at low concentrations, exhibit low toxicity, and do not cause skin discoloration [92]. Furthermore, introducing them into the wound microenvironment lowers the mitochondrial membrane potential and initiates neutrophil apoptosis leading to a reduction in cytokine production. This, consequently, modulates or reduces the inflammatory response and accelerates the healing process [93].

**Fig. 4:**
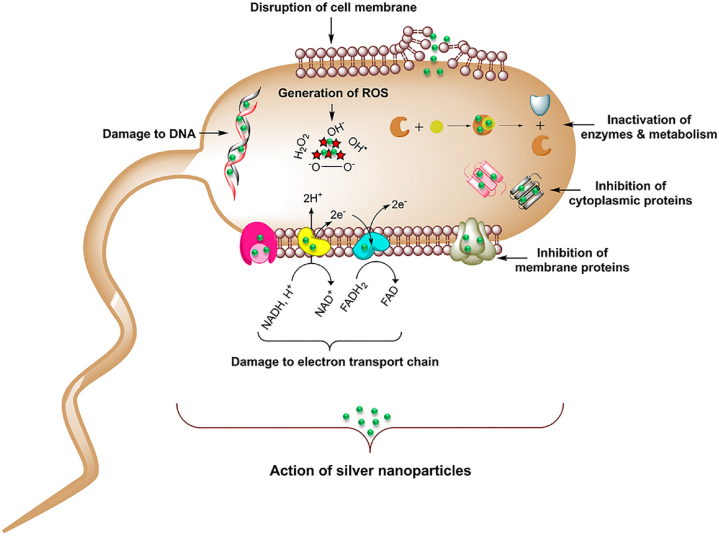
Schematic illustration of the proposed mechanisms of action of AgNPs on bacteria cell apoptosis including destruction of cell membrane, destabilization of structural protein, inactivation of membrane protein, enzyme inactivation, inhibition of electron transport chain, damage to nucleic acids, and oxidative stress stemmed from ROS generation [100] (Published by the Nature Publishing Group (NPG) under the terms of the Creative Commons Attribution License http://creativecommons.org/licenses/by/4.0/).

The antibacterial activity of AgNPs against *E. coli*, *S. aureus*, *methicillin-resistant S. aureus (MRSA)*, and *Pseudomonas aeruginosa* has been widely reported in the literature. As an example, AgNPs form holes in *E. coli* cell walls and accumulate in membranes leading to bacterial death. AgNPs’ antibacterial effects help destroy pathogenic microbes that disrupt regular stages of the wound healing process [[94], [95], [96], [97]]. As suggested in the literature, hypothetical mechanisms for AgNPs' antibacterial characteristics include (i) accumulation of NPs in the microbial membrane which in turn alters microbial permeability and subsequent release of membrane proteins, lipopolysaccharides, and intracellular biomolecules, (ii) NPs-mediated generation of ROS leading to the oxidative degradation of cells, and (iii) metabolization of NPs by microorganisms, reduction in intracellular levels of adenosine triphosphate (ATP) and disruption of DNA replication [98,99]. The antimicrobial functions of AgNPs are schematically shown in Fig. 4.

AgNPs have been integrated into a variety of wound dressings with various morphologies such as nanofiber, hydrogel, and semipermeable membrane [20]. Typically, they are incorporated into the polymeric matrices, colloids, and gels to create an antibacterial nanocomposite efficient for wound dressing. Many other groups of materials are also admixed with AgNPs, such as amino acids, proteins, alkaloids, and phenols, to provide assistance in the reduction and stabilization of NPs and synergistically promote the healing process [52]. Chitosan and gelatin are among the most frequently used natural polymers with which the AgNPs have been formulated [[101], [102], [103]]. More recently, Sudhakar et al. [104] synthesized gelatin-silver nanocomposite hydrogel wound dressings via a simple one-step method using Schiff base bonding and lactose as a crosslinker. Montaser et al. [105] designed AgNPs containing nonwoven fabrics and investigated the effect of their concentration as well as coating of the dressings with carboxymethyl chitosan, alginate, and gelatin on their antibacterial performance. They concluded that the concentration of the absorbed NPs is proportionately associated with its content in the colloidal solution during the padding process. As mentioned earlier, dressings with coatings of the polymers demonstrated higher antibacterial efficiency against *E. coli* and *S. aureus*, and more rapid wound recovery in rats compared to the bare dressings.

However, there are two challenges to be addressed associated with the development of AgNPs-containing wound dressings as follows: (i) aggregation in colloidal solutions and (ii) oxidative cytotoxicity towards human cells [106]. In an effort to tackle these hurdles, AgNPs have been incorporated into various polymer matrices such as sponge-like chitosan [107], chitosan/Bletilla striata polysaccharide composited microneedles [108], chitosan hydrogels [109], hybrid chitosan-silk fibroin nanofibers [110], cellulose [106], poly(vinyl alcohol)/sodium alginate hydrogels [111], plasma-treated electrospun polycaprolactone scaffold [112], collagen/chitosan hybrid scaffolds [113], polyurethane foam [114], and so forth. Although these polymers enhance the dressings' wound recovery performance, they deteriorate its thermal stability, mechanical strength, biodegradability, and washing resistance. Therefore, the recent studies on AgNPs-based wound dressings are more focused on addressing these drawbacks. AgNPs’ contribution in healing process and their antibacterial functions in recently-developed wound dressings are represented in Table 2.

**Table 2:** Nanotechnology-driven advantages, antibacterial function of AgNPs, and their effects on the accelerated wound healing mechanisms in advanced dressings.

Formulation	Microstructure properties	Nanotechnology-driven advantages	NPs antibacterial function	NPs function in accelerated wound healing mechanism	Ref.
PVA/Chitosan oligosaccharide-AgNPs electrospun nanofibers	AgNPs particle diameter: 15.31 ± 4 nm; Fibers diameter: 138.16 ± 32.05 nm	Use of nanofibers encourages the growth and attachment of human skin fibroblasts; Nanofiber therapy stimulates the regeneration of collagen by increasing hydroxyproline levels.	Nanofibrous dressings demonstrate wide-ranging antibacterial effectiveness, permeability, and substantial porosity.	Stimulation of the TGFβ1/Smad signaling pathway	[[115], [116], [117]]
Gelatin/chitosan/AgNPs porous sponge composite	AgNPs particle diameter: 2–10 nm; Interconnected pore size: 100–250 μm	Concentration of AgNPs correlates with an increase in porosity, liquid absorption capacity, and absorption rate; Including AgNPs results in elevated tensile strength and elongation at break; The presence of AgNPs leads to a slight decrease in moisture retention due to the agglomeration of NPs.	Inclusion of AgNPs enhances antibacterial activity; With an increase in AgNPs concentration, the antibacterial capacity also rises; Antibacterial efficacy against *E. coli* surpasses that against *S. aureus*, attributed to the higher peptidoglycan content in the cell wall of *S. aureus* compared to *E. coli*; High concentrations of AgNPs exhibit cytotoxicity, as indicated by the MTT assay.	In vivo tests on New Zealand white rabbits infected with *S. aureus* reveal that dressings containing AgNPs demonstrate improved wound shrinkage and complete coverage of new skin with uniform and thick epithelial tissue after 10 days.	[101]
Alginate, gelatin, and carboxymethyl chitosan coated nonwoven fabrics containing AgNPs	AgNPs particle diameter: 10–20 nm	AgNPs inclusion and polymer coatings lead to an increase in swelling ratio and water retention capability of nonwoven fabrics due to the formation of hydrogen bonding between the polymer chains and water molecules; AgNPs inclusion and polymer coatings result in an increase in water vapor transmission while a reduction in tensile strength; Incorporation of AgNPs improves the wound healing process based on wound area observations. AgNPs inclusion and polymer coatings result in an increase in water vapor transmission while a reduction in tensile strength.	Antibacterial activity increases with the inclusion of AgNPs; Antibacterial capacity increases with AgNPs concentration; Antibacterial activities against *S. aureus* were greater than those against *E. coli* due to the ability of AgNPs to block respiratory enzyme pathways and alternate DNA and cell walls in G+>G- bacteria; Untreated AgNPs containing samples exhibit higher efficacy in reducing the growth rate of *E. coli* bacteria compared to the coated samples.	–	[105]
Iturin-AgNPs-based chitosan composite sponge	Iturin-AgNPs particle diameter: 20 ± 10 nm; BET surface area: 8.158 ± 1.245 m^2^/g; Interconnected pore size: 26.589 ± 1.562 nm; Pore volume: 0.051 ± 0.002 cm3/g	An escalation in iturin-AgNPs concentration results in increased porosity, water absorption, and retention capacity.	The inclusion of AgNPs leads to an augmentation in antibacterial activity; Antibacterial capacity rises with the concentration of iturin-AgNPs up to 10 μg/mL, after which it decreases; Enhanced antibacterial activities against *E. coli* are observed compared to those against *S. aureus*.	Positive advancements are noted in the processes of re-epithelialization and collagen formation.	[107]
Silk fibroin-chitosan-AgNPs-curcumin electrospun nanofibers	AgNPs particle size: 354 nm; Fibers diameter: 600–1100 nm	The inclusion of AgNPs results in an expansion of the surface area, consequently leading to higher drug loading and entrapment capacity of nanofibers.	Biocompatibility and cell adhesion, growth, and proliferation of NIH 3T3 fibroblasts are confirmed through the MTT assay; Minimum inhibitory concentration (MIC): 1.04 ± 5.1 mg/mL for *S. aureus* and 0.94 ± 3.6 for *E. coli*	–	[110]
Plasma treated PCL electrospun scaffold coated with AgNPs embedded in gelatin	AgNPs particle size: 9–12 nm; Fibers diameter: 0.8–1.5 μm	The heightened rate of wound healing is positively correlated with an increase in the number of coating times, leading to a greater thickness of the gelatin-AgNPs coating; To safeguard newly formed granule tissue, adhesion between the wound and scaffold decreases as the number of coating times increases.	The thickness of the gelatin-AgNPs coating is directly proportional to the enhancement of antibacterial activity in scaffolds.	–	[112]
Collagen-chitosan-AgNPs hybrid scaffolds	AgNPs particle diameter: 10–25 nm; Interconnected pore size: 136 ± 5 μm; Porosity: 93.6 %	–	Inclusion of AgNPs results in an augmentation of antibacterial activity.; Antibacterial capacity rises with an increase in the concentration of AgNPs; The Minimum Inhibitory Concentration (MIC) is 10 ppm.	AgNPs stimulate both fibroblast migration and myofibroblast differentiation, as indicated by an increased expression of α-smooth muscle actin (α-SMA); Mechanisms involved in wound healing include the regulation of fibroblast migration and macrophage activation.	[113]
(Carbopol-940)-AgNPs hydrogels	AgNPs were synthesized with Mimosa tenuiflora; AgNPs mean particle diameter: 21 nm	The introduction of AgNPs results in an improvement in the wound healing ratio (% contraction) for second-degree burn injuries in the Wistar rats model.	Hydrogels containing AgNPs demonstrate antibacterial activity against both *E. coli* and *S. aureus* strains.	Cellular events occurring during the healing process of second-degree burn injuries encompass inflammation, chemotaxis, angiogenesis, repair, and interactions with extracellular components; Histopathological observations affirm the absence of noticeable inflammation, intact epidermis, presence of some hair follicles, hyperplasia in the basal stratum, acanthosis, and a distinct desmoplasia zone rich in collagen fibers.	[118]

### 4.2 Au nanoparticles

Contrary to AgNPs, gold NPs (AuNPs) lack antibacterial activity on their own and therefore, are hybridized by other biomacromolecules such as chitosan, gelatin, dextran/polyvinyl alcohol, poly(lactic-co-glycolic acid)/poly(caprolactone), and cotton fabric to be effective in accelerating wound healing process [22,[119], [120], [121]]. Collagen, for example has been employed as a cross-linking agent to help AuNPs combine with other biomacromolecules including polysaccharides, peptides, and growth factors, making them biocompatible and biodegradable to be used as wound dressing [119]. Moreover, AuNPs have been bioconjugated to other nanoparticles, biomolecules, stem cells, and specific antibodies to improve their antimicrobial performance [[122], [123], [124], [125]]. For example, the activity of vancomycin against vancomycin-resistant enterococci and *E. coli* was substantially enhanced when coupled with AuNPs [126]. Besides, it has been revealed that conjugating AuNPs with photosensitizers could improve their antifungal activity against Candida albicans [127].

AuNPs-based in vitro wound healing is accelerated due to their free radical scavenging activity [128]. AuNPs conjugated with chitosan demonstrated significantly higher free radical scavenging activity and enhanced biocompatibility compared to the bare NPs. The inclusion of AuNPs in chitosan-based wound dressing led to enhanced hemostasis, epithelial tissue formation with a high healing rate, and rapid wound closure [129]. In another study, higher wound recovery rate, lower inflammation, and improved collagen deposition were obtained with application of AuNPs-conjugated human fibroblasts on the burnt skin surface [124]. AuNPs’ contribution in healing process and their antibacterial functions in recently-developed wound dressings are represented in Table 3.

**Table 3:** Nanotechnology-driven advantages and antibacterial function of AuNPs along with their effects on the accelerated wound healing mechanisms in advanced dressings.

Formulation	Microstructure properties	Nanotechnology-driven advantages	NPs antibacterial function	NPs function in accelerated wound healing mechanism	Ref.
AuNPs-encapsulated dextran-PVA nanoparticles	Mean NPs size: 10–50 nm	Addition of AuNPs results in a rise in tensile strength, modulus, and rupture elongation in the manufactured nanocomposites; Bandages containing AuNPs display a reduced degradation rate, increased swelling potential (water absorption), and an enhanced rate of wound closure in an in vivo mouse model.	Nanocomposites containing AuNPs demonstrate antibacterial activity against both *E. coli* and *S. aureus* strains, along with sustained cell viability when tested with non-cancerous NIH-3T3 cells.	–	[22]
AuNPs- Pluronic®127-hydroxypropyl methylcellulose thermoresponsive gels	AuNPs particle size:28.9–37.65 nm	Gels incorporating AuNPs demonstrate increased contraction of burn diameter, indicating higher efficacy in wound healing.	Gels containing AuNPs exhibit antibacterial activity against strains of *S. aureus*.	Histopathological examinations confirm focal hyperkeratosis and acanthosis in the epidermis, localized granulation tissue formation with infiltrating inflammatory cells in the dermis, and marked congestion in blood vessels with focal hemorrhage.	[119]
Au nanorods-PLGA-PCL scaffolds	–	–	–	Scaffolds incorporating Au nanorods induce cell proliferation in vitro by generating mild heat through laser irradiation, elevating the local temperature up to 40 °C; Mechanism for accelerated wound healing involves regulating the expression of heat shock protein (HSP70) through external light stimulation.	[120]
AuNPs-coated cotton fabrics	AuNPs particle size:20 nm	An extract containing AuNPs demonstrates antioxidant activity in countering DPPH free radical scavenging; Capacity for radical scavenging increases with the concentration of AuNPs.	Cotton coated with AuNPs displays antibacterial activity against both *E. coli* and *S. aureus* strains; Antibacterial effectiveness against *S. aureus* surpasses that against *E. coli*.	AuNPs contribute to re-epithelialization, collagen deposition, and the coverage of wound areas.	[121]
AuNPs- (cryopreserved human culture of fibroblasts)- methylcellulose gel	Mean size of AuNPs: 15 nm	Addition of AuNPs results in heightened proliferative activity of fibroblast cells, an increased rate of burn area contraction, and an accelerated wound healing process.	–	Gels containing AuNPs expedite wound healing by enhancing fibroblast cell proliferation, regulating collagen synthesis/degradation, and modifying the composition of type I and III collagen in the wound area.	[124]
AuNPs/AgNPs containing wool fat-paraffin-cetostearyl alcohol ointment	AuNPs and AgNPs particle sizes: 5–15 nm and 5–18 nm respectively	The formulations display antioxidant efficacy in countering DPPH free radical scavenging; The inhibition percentages for DPPH radical scavenging are as follows: AgNPs 53.7 %, AuNPs 66.2 %, and ascorbic acid 88.2 %.	–	Excision wounds treated with formulations containing AuNPs and AgNPs show accelerated rates of wound closure; Treated open skin wounds exhibit notable formation of granulation tissue, collagen deposition, re-epithelialization, and faster wound closure compared to both the control and the standard drug Soframycin.	[128]

### 4.3 Cu nanoparticles

Copper NPs (CuNPs) have been extensively studied for use in wound healing applications due to their superior antibacterial performance and ability to suppress a broad range of bacterial strains [23,[130], [131], [132], [133], [134], [135], [136], [137], [138], [139], [140], [141], [142]]. Release of Cu ions from these NPs causes cell wall disruption by stiffening protein structure or changing the enzymatic function. The antibacterial potency of CuNPs depends considerably on their size and concentration, with their complete inhibitory effect on *E. coli* at higher concentrations. CuNPs with sizes of 40 nm and 80 nm were found to be non-lethal to the skin at the concentrations of 10 μM and 1 μM, respectively. CuNPs have demonstrated the ability to adhere to bacteria and penetrate their cell membranes. The released Cu ions damage the bacterial cell wall and then degrade its cytoplasm leading to bacterial death [131,143,144]. Overproduction of bacterial ROS following the introduction of CuNPs results in the peroxidation of cellular lipids and proteins, DNA degradation, and consequently bacterial death [145,146].

In general, the released Cu ions interact with and modulate the expression and activity of proteins and growth factors involved in various stages of wound healing process. These proteins include platelet-derived growth factor (PDGF) [[147], [148], [149]], vascular endothelial growth factor (VEGF) [[150], [151], [152], [153]], angiogenin (ANG) [[154], [155], [156]], matrix metalloproteinases (MMPs) [157], tripeptide glycyl-L-histidyl-L-lysine (GHK) [[158], [159], [160], [161], [162], [163], [164]], nerve growth factor (NGF) [[165], [166], [167]], integrins [[168], [169], [170]], and Lysyl oxidase [171,172].

Taking advantage of CuNPs' antimicrobial activity, He et al. [173] loaded them into bacterial cellulose (BC) membranes via an in situ chemical reduction method. CuNPs were uniformly distributed and physically anchored throughout the BC nanofibrous network. Long-term antibacterial activity has been observed against *S. aureus* and *E. coli* due to the sustainable release of Cu ions for three months. Of note, it is also necessary to tailor CuNPs’ concentration to suppress its cytotoxicity in the BC matrix.

In addition to BC and natural cellulose [134], CuNPs-containing chitosan and gelatin-based scaffolds have also been developed for wound dressing applications. Kumari et al. [174] investigated the effects of CuNPs on physicochemical properties of chitosan and gelatin composite scaffolds fabricated by a freeze-drying method. They concluded that CuNPs-loaded scaffolds bear an appropriate porosity and pore size, cell viability, hemocompatibility, cytocompatibility, and degradation rate to be used in wound healing applications and skin tissue engineering. More recently, Glushchenko et al. [132] reported that adding Cu/CuOx NPs and chitosan/chitosan derivatives into cellulose-based hydrogel ointment increases wound closure rate and provides efficient angiogenesis, re-epithelisation, collagen deposition in the wound, antibacterial activity and cell biocompatibility. Accordingly, CuNPs activate the signaling pathways related to wound repair regulation which may involve iNOS, a family of enzymes that catalyze NO production. NO interacts with superoxide anions inside the wound to produce peroxynitrite. They function as pro-oxidants and stimulate the body’s antioxidant system. This reduces the concentration of superoxide anions and increases endogenous NO, ultimately accelerating regeneration and wound contraction. Interestingly, the presence of Cu and Au hydroxide in the cow urine gives it an antitoxic function making it a useful substrate for wound healing and is therefore used to eliminate the toxic effects of drug residues [175]. CuNPs' applications in wound healing process and their antibacterial functions in recently-developed wound dressings are represented in Table 4.

**Table 4:** Nanotechnology-driven advantages and antibacterial function of CuNPs along with their effects on the accelerated wound healing mechanisms in advanced dressings.

Formulation	Microstructure properties	Nanotechnology-driven advantages	NPs antibacterial function	NPs function in accelerated wound healing mechanism	Ref.
CuNPs	CuNPs particle sizes: 20 nm, 40 nm, 80 nm	Fibroblast and keratinocyte cell viability remains unaffected by exposure to CuNPs, concurrently fostering the migration of HDF, HEK, and HUVEC cells. Furthermore, CuNPs promote endothelial and fibroblast cell proliferation, collagen deposition, and facilitate skin wound healing.	–	Cultured fibroblast cells demonstrate increased expression of collagen 1A1 and enhanced neovascularization due to the presence of CuNPs; Wounds treated with CuNPs predominantly display granulation dermis and skin adnexa, encompassing sebaceous glands and hair follicles.	[143]
CuNPs containing bioactive glass nanocoating on natural eggshell membrane	Eggshell membrane has a porous fibrous structure; Nanocoating thickness: 40–50 nm	The application of a nanocoating containing CuNPs significantly enhances the hydrophilicity and surface hardness of the eggshell membrane.	CuNPs-containing nanocoating increases the antibacterial activity of eggshell membrane against *E. coli*; The antibacterial capacity rises with the concentration of CuNPs.	Stimulation of proangiogenesis is achieved by enhancing the secretion of vascular endothelial growth factor (VEGF), hypoxia-inducible factor (HIF)-1a protein, and the expression of angiogenesis-related genes (VEGF, HIF-1a, VEGF receptor 2 (KDR), and endothelial nitric oxide (NO) in human umbilical vein endothelial cells (HUVECs); In vivo, there is an improved healing quality evidenced by significantly enhanced angiogenesis indicated by CD31 expression and the formation of a continuous and uniform epidermis layer; The sustained release of Cu2+ ions from the nanocoating promotes both angiogenesis and antibacterial activity.	[176]
CuNPs coated fibrous natural cotton-based cellulose	CuNPs particle size: below 5 nm	–	Cotton coated with CuNPs demonstrates high effectiveness in eradicating A. baumannii, a multidrug-resistant bacterial pathogen. This efficacy is attributed in part to a contact-killing mechanism and an augmented release of metal ions.	–	[134]
CuNPs-PVP-HPMC hydrogels	CuNPs particle size: 50, 150 nm	The wound closure rate is enhanced by 12.27 % in hydrogels containing CuNPs; Achieving complete wound closure by the 14th day indicates accelerated wound healing.	–	The hydrogel demonstrates a notable decrease in pro-inflammatory cytokines (IL-6 by 39.74 % and TNF-α by 49.37 %) and an elevated level of anti-inflammatory cytokines (IL-10 by 30.90 %), signifying a reduction in inflammation.	[135]
CuNPs-bacterial cellulose membranes	CuNPs particle size: 10, 1000 nm; Uniform distribution and physical anchoring of CuNPs throughout the BC nanofibrous network.	BC membranes containing CuNPs display prolonged antibacterial activity, releasing Cu ions continuously for 90 days after immersion; CuNPs-incorporated BC membranes show no apparent cytotoxicity towards normal human dermal fibroblasts (NHDF).	The sustained release of Cu ions from CuNPs-containing BC membranes demonstrates antibacterial effectiveness against both *S. aureus* and *E. coli*; CuNPs concentration in the BC matrix should be adjusted to mitigate its cytotoxic effects; The antibacterial efficacy against *S. aureus* surpasses that against *E. coli*.	–	[173]
CuNPs-chitosan-gelatin porous scaffolds	CuNPs mean size: 5.31 nm; Interconnected pore size: 25–40 μm and 95 μm before and after CuNPs inclusion, respectively. • Porosity: 65–88 %	Scaffolds containing CuNPs exhibit high hemocompatibility; The addition of CuNPs to the polymer matrix enhances in-vitro biodegradation rates, with no significant impact on cell adhesion and proliferation.	–	–	[174]
N, N‐bis(acryloyl) cystamine (BACA) chelated CuNPs/methacrylate modified gelatin (Gel‐MA) hydrogels	CuNPs mean diameter: 88 nm	The incorporation of CuNPs into hydrogels results in an increased degradation rate, reduced swelling ratio, elevated wound closure rate, and temperature elevation under NIR laser irradiation. However, lipid peroxidation of bacteria is not significantly affected.	Hydrogels containing CuNPs display notable antibacterial activity against both *S. aureus* and *E. coli*, attributed to the sustained release of Cu ions; CuNPs-embedded hydrogels demonstrate improved antibacterial efficacy when subjected to NIR laser irradiation, owing to accelerated Cu ion release and their photothermal performance, generating localized heat through the localized surface plasmon resonance (LSPR) effect.	In an infected chronic wound model with *S. aureus*, accelerated healing mechanisms involve stimulated NIH-3T3 fibroblast proliferation through Cu ion release, enhanced antibacterial capability, reduced inflammatory response, and promoted angiogenesis ability in vivo; No alterations were observed in IL-1β , IL-6, TNF-α, and IL-10 expressions.	[177]

### 4.4 Advantages and disadvantages of MNPs in wound dressings

MNPs have emerged as promising candidates for the development of advanced wound dressings due to their unique physicochemical properties. Their small size, large surface area, and antimicrobial activity make them attractive for various biomedical applications. However, a comprehensive understanding of their advantages and disadvantages is crucial for their safe and effective implementation.

#### 4.4.1 Advantages of MNPs in wound dressings

One of the primary advantages of MNPs is their potent antimicrobial activity. Several studies have demonstrated the efficacy of MNPs in combating a wide spectrum of bacteria, fungi, and viruses [178,179]. This broad-spectrum antimicrobial action is particularly beneficial in the prevention and treatment of infections, which can significantly impair wound healing [180].

Moreover, MNPs can enhance wound healing by promoting cell proliferation, migration, and angiogenesis. Certain MNPs such as CuNPs, have shown anti-inflammatory properties, which can aid in reducing inflammation and promoting tissue regeneration. Additionally, some MNPs possess catalytic properties that can facilitate the breakdown of wound debris and promote wound debridement [54,181,182].

#### 4.4.2 Disadvantages of MNPs in wound dressings

Despite their promising properties, MNPs also present certain challenges. A primary concern is their potential cytotoxicity. While some MNPs, such as AuNPs, are generally considered biocompatible, others, like AgNPs and CuNPs, can exhibit toxicity at higher concentrations or prolonged exposure. This necessitates careful optimization of NPs concentration and formulation to ensure safety and efficacy [[183], [184], [185]].

Another challenge is the potential for nanoparticle aggregation and agglomeration, which can reduce their antimicrobial activity and affect their overall performance. The stability of MNPs-based wound dressings in the complex wound environment is crucial for maintaining their therapeutic efficacy [186,187].

Furthermore, the long-term effects of MNPs exposure on human health and the environment are still under investigation. The potential for nanoparticle release into the environment and its impact on ecosystems is a growing concern [188,189].

#### 4.4.3 Comparative analysis of MNPs

While different types of MNPs share some common advantages and disadvantages, there are also significant variations in their properties. For instance, AgNPs exhibit strong antimicrobial activity but can be cytotoxic at higher concentrations, whereas AuNPs are generally considered biocompatible but have lower antimicrobial efficacy. CuNPs offer a balance between antimicrobial activity and cost-effectiveness but may cause tissue staining [[190], [191], [192]].

Understanding these differences is crucial for selecting the most appropriate MNPs for a specific wound healing application. It is important to consider factors such as the type of wound, the target pathogen, and the desired therapeutic outcome when choosing a MNPs-containing wound dressing. In conclusion, MNPs have the potential to revolutionize wound care. However, careful consideration of their advantages and disadvantages is essential for the development of safe and effective wound dressings. Future research should focus on addressing the challenges associated with MNPs toxicity, stability, and environmental impact while exploring new strategies for enhancing their therapeutic efficacy.

## 5 Metal oxide nanoparticles

### 5.1 ZnO nanoparticles

ZnONPs’ remarkable antibacterial properties have led to their widespread use in wound dressing [[193], [194], [195]]. As a primary mechanism, antibacterial activity of ZnONPs stems from the generation of free hydroxyl radicals using ultraviolet photocatalysis which in turn disrupts the cell membrane, leading to bacterial death. ROS generation accelerates the process. It is worth noting that the antibacterial activity of ZnONPs is inversely correlated with their size with smaller size of NPs, showing higher antibacterial activity. This is due to the accumulation of higher quantity of smaller NPs in the cytoplasm and cell membrane [[196], [197], [198], [199]]. ZnONPs have been extensively embedded into wound dressing matrices to enhance their antimicrobial activity and wound recovery performance. These matrices include but may not limited to alginate hydrogels [197] and electrospun membranes [200], collagen dressings [201], chitosan hydrogels [202], chitosan/poly (ethylene glycol) nanocomposite [203], carboxymethyl cellulose sheets [204], chitosan/glycogen/ZnONPs-doped cotton pads [205], AgNPs/ZnO containing polyurethane nanofibers [206], poly(lactide-co-glycolic acid) (PLGA)/silk fibroin electrospun membranes [207], chitosan/poly(vinyl alcohol)/heparinized ZnONPs hydrogels [208], BC/betulin diphosphate nanocomposites [209], hydrocolloid dressings [33], collagen blended ZnONPs embedded niosome nanocomposites [210], polyacrylic acid/polyallylamine hydrochloride electrospun fibers [211], thixotropic xymedone gels [212], alginate/gum acacia nanocomposites [213].

Aly et al. [214] designed cellulose acetate nanofibers containing ZnONPs, graphene oxide (GO), and ZnONPs/GO for wound healing. They reported that incorporating ZnONPs and GO into the nanofibers improves surface roughness, tensile strength, toughness, cell viability, and human fibroblasts cell adhesion. Saddik et al. [215] used ZnONPs as drug carriers for treatment of infected wounds. Azithromycin (AZM)-loaded ZnONPs showed more effective antimicrobial activity than control samples. Impregnation of AZM-ZnONPs into hydroxyl propyl methylcellulose gel leads to an enhanced bacterial clearance and epidermal regeneration and stimulates tissue formation. Many other NPs have been hybridized with ZnONPs to promote their wound-dressing qualities. For instance, Sirotkin et al. [216] synthesized ZnO, TiO_2_, and Zn/TiO_x_ NPs via an underwater pulse discharge plasma and investigated their antimicrobial effectiveness against *E. coli*, S. albicans, and B. subtilis as wound-healing viscose patches. They reported that the antimicrobial activity of viscose patches containing 0.22 % Zn/TiOx NPs was higher than that of two separately-added NPs. ZnONPs’ properties in healing process and their antibacterial functions in recently-developed wound dressings are represented in Table 5.

**Table 5:** Nanotechnology-driven advantages and antibacterial function of ZnONPs along with their effects on the accelerated wound healing mechanisms in advanced dressings.

Formulation	Microstructure properties	Nanotechnology-driven advantages	NPs antibacterial function	NPs function in accelerated wound healing mechanism	Ref.
ZnONPs-CuONPs- gelatin- hyaluronic acid-chondroitin sulfate-Asiatic acid hydrogels	ZnONPs & CuNPs size >50 nm	Hydrogels containing ZnONPs and CuONPs demonstrate a gradual increase in L929 cell proliferation from day 1 to day 3 in the MTT assay.	ZnONPs and CuONPs embedded in hydrogels display antibacterial efficacy against both *S. aureus* and *E. coli*, attributed to the gradual release of nanoparticles.	Hydrogels exhibit a higher rate of wound contraction compared to cotton gauze, similar to NeuSkin™; Serum biochemistry analysis indicates no toxicity or inflammation in the rats; Histopathology studies reveal (i) re-epithelization and collagen bundle formation, and (ii) reduced inflammation and enhanced tissue remodeling evidenced by a decrease in TNF-α activity and an increase in MMP-2 marker on the 7th day of the experiment.	[217]
ZnONPs-Carbopol 940 hydrogels	ZnO nanowires diameter: <100 nm	The incorporation of green ZnONPs into hydrogels results in an elevated wound closure rate and accelerated wound healing, manifested through enhanced epithelization, vasculature, reduced necrosis, connective tissue formation, and increased collagen synthesis in full-thickness skin wounds.	–	On the 3rd day, observations reveal necrotic tissues with a small blood clot, edema, and substantial infiltration of inflammatory cells, predominantly neutrophils; By the 7th day, there is a predominant proliferation of fibroblasts accompanied by the rich production of collagenous fibers, high numbers of cellular macrophages, and lymphocyte infiltration, along with the formation of newly-developed blood vessels; On the 14th day, significant re-epithelization of granulation tissue is noted, with reduced inflammatory cell infiltration and abundant collagenous fibers; By the 21st day, complete reepithelization is observed, with an intact epidermis, minimal inflammatory cell infiltration, the presence of newly-formed blood vessels, and the formation of a mature and rich scar.	[193]
ZnO, Ag, and Ag/ZnO-NPs impregnated cotton bandages	Mean diameters of green synthesized ZnONPs and AgNPs: 26 and 16 nm, respectively	–	Bandages containing ZnONPs, AgNPs, and a combination of both types of nanoparticles demonstrated antibacterial efficacy against Acinetobacter baumannii and *Pseudomonas aeruginosa*; The antimicrobial impact of AgNPs was marginally superior to that of ZnONPs and a combination of Ag/ZnO-NPs.	–	[194]
Core-shell hyaluronic acid (HA)-silk fibroin (SF)-ZnONPs nanofibers	ZnONPs diameter: <50 nm; Mean fiber diameter: from 155 ± 11 nm for HA–SF to 189 ± 15 nm for HA–SF/(5 % w/v) ZnONPs	In in-vitro cytotoxicity testing, nanofibers with a ZnONPs concentration exceeding 3 wt% demonstrate toxicity to cells; In in vivo experiments, nanofibers containing 3 wt% ZnONPs facilitate wound healing and notably reduce the inflammatory response; Wettability, indicated by a water contact angle of 77.45°, and the in-vitro degradation of nanofibrous scaffolds remain unaffected by the ZnONPs content.	Nanofibers containing ZnONPs exhibit antibacterial activity against both *S. aureus* and *E. coli*.	ZnONPs-containing nanofibers enhance wound healing by promoting cell attachment, proliferation, and viability in HaCat cell culture.	[218]
Chitosan-(Ag-doped) ZnONPs membranes	ZnONPs mean diameter: 52 nm; Ag-doped ZnONPs mean diameter: 37 nm	The band gap for ZnONPs synthesized using a green method is 3.39 eV, while for Ag-doped ZnONPs, it is 3.25 eV; The antidiabetic activity was assessed through α-amylase and α-glucosidase inhibition assays at concentrations of 100 μg/mL and 200 μg/mL; Membranes containing ZnONPs and Ag-doped ZnONPs exhibit enhanced wound healing potential, resulting in a more substantial reduction in wound area; The introduction of Ag into ZnONPs promotes the wound healing capacity of the membranes.	Membranes containing Ag-doped ZnONPs demonstrate antibacterial efficacy against cocci and *E. coli*; The antibacterial effectiveness against cocci surpasses that against *E. coli*, with an inhibition zone of 19 mm compared to 16 mm, attributed to the thinner and impermeable peptidoglycan layer of gram-negative bacteria; The antibacterial activity of Ag-doped ZnONPs arises from the strong electrostatic interaction between Zn and Ag ions and the negatively charged outer surface of bacteria.	–	[195]

### 5.2 TiO_2_ nanoparticles

Green synthesized TiO_2_NPs exhibit remarkable wound recovery capabilities, primarily due to their effectiveness in wound closure, as well as their histopathology, and protein expression profiling [[219], [220], [221], [222], [223]]. TiO_2_NPs are introduced into the wound dressing media such as chitosan [[224], [225], [226], [227], [228]], polyurethane [229,230], xylan/chitosan [231], poly(vinyl alcohol)/chitosan-g-poly (N-vinyl imidazole) [232], chitosan/maleic terminated polyethylene glycol [233], polycaprolactone-chitosan [234], polyvinyl alcohol/sodium alginate [235], gellan gum [236,237], gelatin [238], cellulose acetate [239], silk fibroin/collagen [240], heparin-polyvinylpyrrolidone [241], human amniotic membrane [242], and carrageenan [243], to promote their mechanical, antimicrobial, and biocompatibility properties.

Moreover, TiO_2_NPs are combined with other nanomaterials such as glucose oxidase/Fe_3_O_4_/Ag_3_PO_4_ [143], BaTiO_3_/AuNPs [144], and GO. Prakash et al. [239], for example, synthesized cellulose acetate nanofibers containing GO/TiO_2_/curcumin for wound healing application. The NPs containing electrospun nanofibers exhibited an enhanced antibacterial activity, tensile strength, swelling, degradation rate, hemocompatibility, cell proliferation, and migration compared to pure cellulose acetate nanofibers. In another study, Venkataprasanna et al. [244] fabricated a chitosan/poly (vinyl alcohol)/GO loaded with vanadium-doped TiO_2_ patch for visible light-driven antibacterial activity and promoted wound healing applications. They reported that the patch could provide an excellent moist environment for wound breathing owing to its high surface porosity, appropriate moisture vapor transfer rate, desirable swelling behavior, and oxygen permeability. It also exhibited significantly elevated antibacterial activity and biocompatibility favorable to cell growth and differentiation. TiO_2_NPs’ contribution in healing process and their antibacterial functions in recently-developed wound dressings are represented in Table 6.

**Table 6:** Nanotechnology-driven advantages and antibacterial function of TiO_2_NPs along with their effects on the accelerated wound healing mechanisms in advanced dressings.

Formulation	Microstructure properties	Nanotechnology-driven advantages	NPs antibacterial function	NPs function in accelerated wound healing mechanism	Ref.
ZnONPs, TiO2NPs, and mixed Zn/TiO_x_‐NPs impregnated viscose patch	TiO_2_NPs average size: 120 nm; ZnONPs particle size: 30–250 nm; Zn/TiO_x_‐NPs average size: 90 nm	–	Created viscose patches demonstrate antibacterial efficacy against *E. coli*, S. albicans, B. subtilis, and C. albicans; The most potent antimicrobial activity is observed in the viscose patch containing 0.22 % Zn/TiO_x_‐NPs; The mechanisms underlying the antibacterial activity involve the release of antibacterial ions Zn^2+^ and Ti^4+^, interaction between nanoparticles and microorganisms, consequent destruction of bacterial cell walls, and the generation of ROS.	–	[216]
Green synthesized TiO_2_NPs	TiO_2_NPs average particle size: 320 nm	The application of green TiO_2_NPs accelerates wound healing in Albino rats, as evidenced by enhanced wound closure, improved histopathology, and altered protein expression profiling.	–	The use of TiO_2_NPs results in healing without pus formation, bleeding, microbial infections, and persistent inflammation; The enhanced wound closure facilitated by TiO_2_NPs is attributed to their abundant availability, which promotes increased fibroblast proliferation and collagen activity in regenerated wound tissues; TiO_2_NPs stimulate the aggregation of macrophages, proliferation of fibroblasts, collagen deposition, and increased cellular infiltration and epithelialization; Protein expression analysis of animals treated with TiO_2_NPs reveals notable differential expression in proteins and an increase in the densities of many bands, accompanied by a decrease in densities of some other bands.	[219]
TiO_2_NPs	TiO_2_NPs particle size: 10, 15, 25, 50 nm	TiO_2_NPs enhance fibroblast cell adherence, proliferation, and migration; Cell proliferation is similarly boosted by TiO_2_NPs of different sizes and phases, including rutile and anatase.	Synthesized TiO_2_NPs display antibacterial activity against *Salmonella enterica* Typhimurium, *E. coli*, *S. aureus*, and B. subtilis; The antibacterial activity increases with decreasing nanoparticle size; TiO_2_NPs attach to rigid and unimpaired bacterial cell walls through particle/cell wall electrostatic interaction, leading to distortion, plasmolysis, and cell wall damage. The cell ultimately succumbs to increased oxidative stress and cytoplasmic leakage; Smaller TiO_2_NPs, with higher surface area, exhibit increased solubility, releasing more Ti ions into the medium. This results in higher cell wall interaction and attachment, increased accumulation inside the cell, leading to elevated ROS generation, and subsequent DNA damage or inactivation of cellular proteins.	TiO_2_NPs inhibit bacterial growth at the wound site; They promote the production of critical growth factors for wound healing, including endothelial growth factor, platelet-derived growth factor, Monocyte chemoattractant protein (MCP)-1, Macrophage inflammatory protein (MIP)-1, pro-inflammatory chemokines Interleukin (IL-8), and MIP-1; TiO_2_NPs contribute to enhanced blood clotting; The process involves fibrin formation and platelet activation, leading to the release of growth factors and subsequent activation of neutrophils and monocytes.	[223,245]

### 5.3 Iron oxide nanoparticles

Iron oxide NPs are among the most promising nanomaterials for biomedical applications, particularly drug delivery and wound repair [[246], [247], [248]]. Ziv-Polat et al. [249,250] utilized Fe_2_O_3_NPs to stabilize thrombin as a topical hemostatic agent and examined the wound healing efficiency of the Fe_2_O_3_NPs bound thrombin compared to free thrombin. It was revealed that thrombin-conjugated γ-Fe_2_O_3_NPs offer a remarkably accelerated wound healing and higher tensile strength of the regenerated skin. The stabilized thrombin could also enhance adhesion, remove skin grafts, and facilitate the removal of earlier sutures, which in turn lowers the risk for wound dehiscence and stitch-induced scarring.

**Fig. 5:**
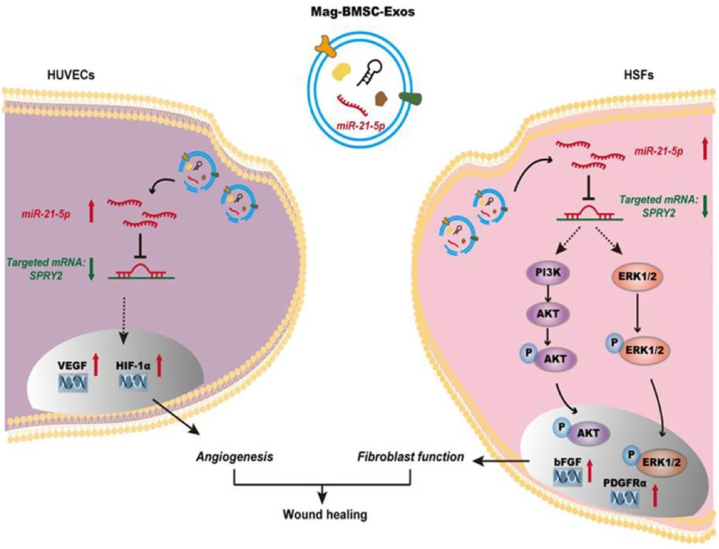
Schematic illustration for the mechanism of action of Fe_2_O_3_NPs/BMSC-Exos into rat skin wounds (Reprinted with permission from Ref. [253]. Copyright 2023 Taylor & Francis Online).

The application of magnetic NPs and a persistent low magnetic force on cells in a static magnetic field has the potential to stimulate tissue regeneration through regulating the phenotypic polarization of fibroblasts [251,252]. Wu et al. [253] utilized exosomes derived from bone mesenchymal stem cells (BMSC-Exos) in combination with magnetic Fe_2_O_3_NPs to synthesize a specific wound dressing. They reported that Fe_2_O_3_NPs/BMSC-Exos administration significantly improved cell proliferation, migration, and angiogenesis compared to the administration of BMSC-Exos alone. Local transplantation of Fe_2_O_3_NPs/BMSC-Exos into rat skin wounds resulted in accelerated wound closure, constricted scar dimensions, and enhanced angiogenesis and fibroblast function due to miR-21-5p upregulation, employing inhibiting SPRY2 and activating PI3K/AKT and ERK1/2 signaling pathways. Fig. 5 provides an illustration of this possible mechanism.

Superparamagnetic iron oxide NPs (SPIONs), which consist of a Fe^3+^ and Fe^2+^ ion core and an outer layer of dextran or another polysaccharide coating, are also widely used in wound dressing owing to their extraordinary antibacterial and physicochemical properties [[254], [255], [256]]. SPIONs-decorated carbon nanotubes prepared by pulsed laser ablation method have demonstrated antibacterial activity against different strains of bacteria in animal models [257]. SPIONs are also coated with certain biopolymers, such as polyethylene glycol and dextran to facilitate the conjugation of therapeutic components and enhance their distribution in bloodstream are [256]. Wu et al. [258] used SPIONs modified by dopamine heparin conjugate (HDC) to stabilize the fibroblast growth factor (bFGF) and provide its prolonged release in the wound environment. bFGF-HDC@Fe_3_O_4_ NPs promote wound recovery through polarization of M2 macrophage and enhanced cell proliferation. Ratwa et al. [158] studied the effects of SPIONs-impregnated BC on the mechanical and physicochemical characteristics of alginate/casein injectable porous hydrogel for wound healing applications. They reported higher compression strength, increased porosity and swelling, and improved cell viability and drug release behavior for the hybridized gels.

## 6 MXene nanoparticles

**Fig. 6:**
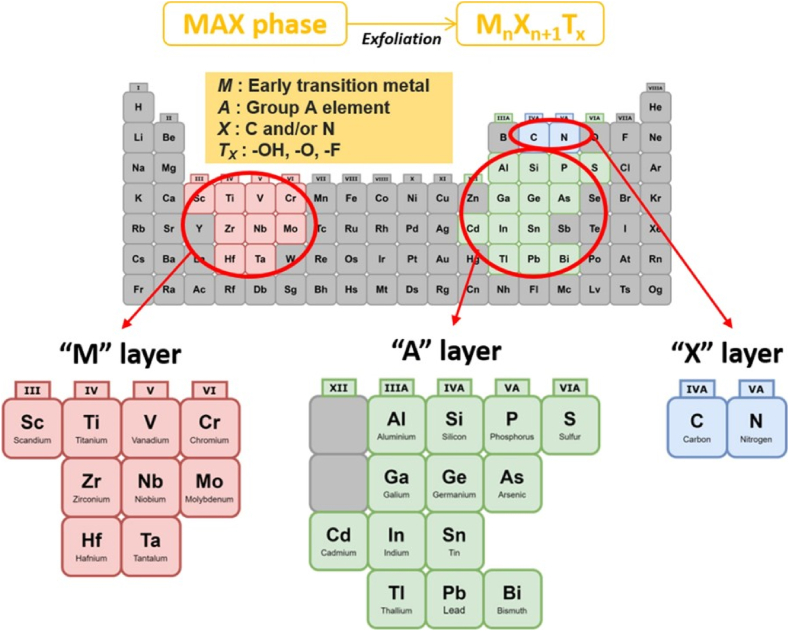
General element composition of MAX phase and MXene [262] (Published by the American Association for the Advancement of Science (AAAS) under the terms of the Creative Commons Attribution License http://creativecommons.org/licenses/by/4.0/).

MXenes are a novel family of two-dimensional nanosheets with customizable properties and multilayer structure made of conical scrolls of transition metal carbides, nitrides, and carbonitrides. They can be represented with the general formula M_n+1_X_n_, where M represents transition metals (e.g., Sc, Ti, Cr, etc.) and X is carbon and/or nitrogen. MXene are synthesized as a result of etching from the MAX phase which is a layered ternary carbide/nitride with the general formula M_n+1_AX_n_, where A represents periodic table of elements groups 13 and 14 (Fig. 6). As an emerging class of nanomaterials, they are frequently synthesized by an exfoliation method at room temperature [[259], [260], [261]]. MXene possess many unique physicochemical properties including: (a) MXene have hydrophilic functional groups on their surface (-F, -OH, -O, etc.), which gives them an advantage as they do not require complex surface modification compared to other hydrophobic NPs; (b) MXene possess high metallic conductivity; and (c) MXene have excellent biocompatibility, allowing it to be removed and degraded in vivo [262].

MXene nanosheets have been recently at the center of attention for their potential use in biomedical applications, particularly wound healing. This was mainly owing to their distinct characteristics including large specific surface area, prominent electrical and antibacterial performance, and superior biodegradability and photothermal properties. While Ti_3_C_2_ and Ti_2_C MXenes are commonly studied, exploring other combinations of elements offers potential for further innovation in biomedical and biotechnology applications [[262], [263], [264], [265], [266], [267], [268], [269]].

In recent years, a miscellaneous assortment of MXenes-integrated wound dressing platforms has been developed to employ outstanding characteristics of MXenes nanosheets in tissue engineering and wound healing applications. PAAV-coated MXene-embedded PAN/PVP nanobelt electrospun fibers [270], regenerated-BC/MXene (Ti_3_C_2_T_x_) hydrogel [271], MXene-amoxicillin-PVA nanofibrous membrane [272], MXene-loaded nanofibers/growth factor-dopamine-hyaluronic acid-containing hydrogel core/shell hybrid structure [273], chitosan/MXene-poly(vinylidene fluoride) membrane [274], a nano-platform of growth factor-decorated Ti_3_C_2_ MXene/MoS_2_ bio-heterojunctions [275], laser-guided graphene/MXene/polydimethylsiloxane sensors-integrated wound bandage [276], MXene/spidroin-incorporated microneedle scaffolds [277], Zn-MOF@Ti_3_C_2_T_x_ MXene Schottky junction [278], Ti_3_C_2_T_x_ MXenes@CeO_2_ nanocomposite scaffolds [279], polyvinyl alcohol/MXene/polyaniline hydrogel [280], injectable hydrogel based on hyaluronic acid-graft-dopamine and polydopamine (PDA)-coated Ti_3_C_2_ MXene nanosheets [281], (MXene@PDA)-decorated chitosan nanofibrous mats [282], MXene-incorporated chitin composite sponges [283], MXene@PVA hydrogel [284], accelerated-wound-healing MXene-based Epidermic sensors [285], and MXene-wrapped magnetic colloids and poly(N-isopropyl acrylamide)-alginate dual-network hydrogels [286] are among the recently published research works in the field.

**Fig. 7:**
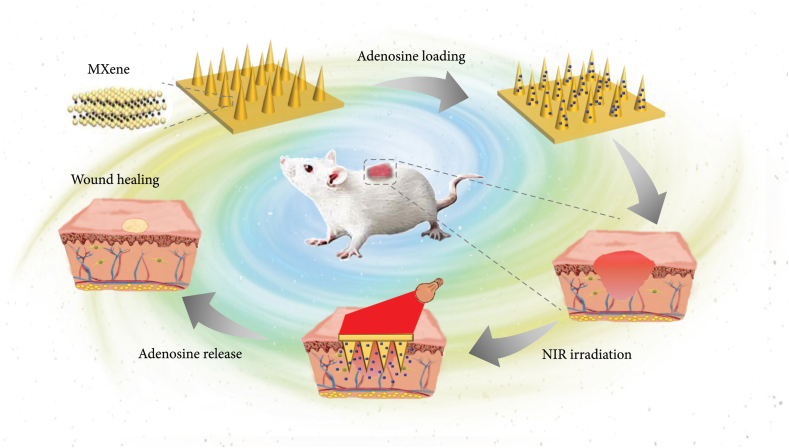
Schematic illustration of the wound healing mechanism for adenosine-encapsulated MXene-integrated microneedle patches [288] (Published by the American Association for the Advancement of Science (AAAS) under the terms of the Creative Commons Attribution License http://creativecommons.org/licenses/by/4.0/).

To overcome the skin tissue’s lack of adhesion, Zhou et al. [287] modified Ti_3_C_2_T_x_ MXenes with PDA and designed a conductive self-healing hemostatic scaffold by cross-linking between branched poly(glycerol-ethylenimine), MXene@PDA, and oxidized hyaluronic acid. The fabricated multifunctional scaffolds displayed tissue adhesion, antibacterial activity, and accelerated healing of wounds infected by methicillin-resistant *S. aureus* through efficient anti-inflammatory effects, promoted cell proliferation and angiogenesis, and stimulated granulation tissue formation. In another study, Sun et al. [288] synthesized MXene-integrated microneedle patches and embedded them into 3-(acrylamido)phenylboronic acid-integrated polyethylene glycol diacrylate hydrogel for wound healing. They encapsulated microneedles with adenosine to benefit from MXenes photothermal conversion capacity. They reported an accelerated release of loaded adenosine under near-infrared irradiation, leading to enhanced angiogenesis (Fig. 7).

While MXenes offer potential for antimicrobial applications, their use as a single agent may have limitations including: (i) inflammation and tissue damage due to the localized high temperature of a single PTT treatment; (ii) lack of sustained antimicrobial activity; and (iii) poor antimicrobial efficacy stemmed from the bacterial self-protection mechanisms. To address these challenges, developing multimodal antimicrobial nanoplatforms that combine MXenes with other therapeutic agents is crucial for enhancing efficacy and reducing adverse effects [272,289,290]. Yang et al. [289] fabricated a PCL-based nanofibrous membrane containing PDA-decorated MXene/Ag_3_PO_4_ bioheterojunctions for wound healing. The nanomaterial exhibits excellent bactericidal properties under NIR light irradiation due to synergistic effects of photothermal therapy, photodynamic therapy, and metal ion therapy. The unique self-rechargeable mechanism allows for sustained antibacterial activity even without continuous light exposure. In vivo studies demonstrate the nanofiber's effectiveness in treating infected wounds by providing a regenerative microenvironment and promoting wound healing processes specially through increased epithelialization, stimulated angiogenesis, and collagen deposition on the wound bed.

MXene-NPs’ effects in healing process and their antibacterial functions in recently-developed wound dressings are represented in Table 7.

**Table 7:** Nanotechnology-driven advantages and antibacterial function of MXene NPs along with their effects on the accelerated wound healing mechanisms in advanced dressings.

Formulation	Microstructure properties	Nanotechnology-driven advantages	NPs antibacterial function	NPs function in accelerated wound healing mechanism	Ref.
Regenerated-BC/MXene (Ti_3_C_2_Tx) hydrogel	Interconnected pore size: 100–500 μm	Composite hydrogels containing Ti_3_C_2_Tx demonstrate outstanding electrical conductivity and biocompatibility, along with desired mechanical properties, flexibility, good biodegradability, thermal stability, and a high water-uptake capacity.	–	When subjected to external electrical stimulation, the hydrogel significantly enhances the proliferation activity of NIH3T3 cells in vitro and expedites the in vivo wound healing process by reducing the wound area; The mechanisms involved include augmented collagen synthesis, vascularization, granulation tissue formation, re-epithelialization, and increased gene expression of growth factors such as VEGF, EGF, and TGF-*β*.	[271]
MXene-amoxicillin-PVA nanofibrous membrane	Mean fiber diameter: 145.35 nm	The temperature of the membranes rises with the duration and intensity of laser irradiation; Nanofibrous membranes demonstrate essential hemocompatibility.	These nanofibrous membranes display outstanding antibacterial efficacy against both *E. coli* and *S. aureus*; The hyperthermia produced by MXene under NIR irradiation hinders bacterial proliferation, enhances the release rate of amoxicillin, and reinforces the antibacterial activity of the membranes.	After NIR irradiation, there is minimal inflammation and increased hair follicle formation with a thickened epidermis.	[272]
Ti_3_C_2_Tx@CeO_2_-polyethylenimine grafted Pluronic F127 and oxidized sodium alginate hydrogel scaffolds	–	Hydrogel scaffolds demonstrate effective injectability, self-healing properties, anti-inflammatory and antioxidative capabilities, conductive bioactivity, tissue adhesion, rapid hemostatic capacity, and a significantly accelerated wound closure rate; The scaffolds exhibit notable anti-inflammatory effects and are effective in multidrug-resistant infection therapy.	Hydrogel scaffolds display outstanding antibacterial efficacy against *E. coli*, *S. aureus*, and MRSA, attributed to the synergistic combination of cationic polyethylenimine and the incorporation of nano-knife-like MXene@CeO_2_ nanocomposites.	The scaffolds promote the migration of fibroblasts and cell proliferation under electrical stimulation; They also enhance fibroblast proliferation, granulation tissue formation, collagen deposition, and re-epithelialization; The mechanisms underlying these effects include: (i) efficient ROS scavenging due to the antioxidative ability of MXene@CeO_2_ nanocomposites; (ii) potent in-vivo anti-infection activity, leading to reduced interleukin-6 (IL-6) and an increased level of IL-10 as an anti-inflammatory cytokine; (iii) increased Ki67 expression indicating accelerated cell proliferation during the middle stage of the healing period; and (iv) increased CD31 expression indicative of stimulated angiogenesis at the middle and late stages of healing.	[279]

## 7 Metal-organic frameworks (MOFs)

MOFs are a group of crystalline coordination polymers of metal ions linked by organic ligands. Studies have been extensively carried out on their applications, especially in biochemistry, biomedicine, bioimaging, and drug delivery, owing to their unique characteristics such as extensive surface area, porous structure with high porosity content, high thermal stability, biocompatibility, biodegradability, and facile synthesis methods. Currently, MOFs have garnered extensive attention in the field of wound healing owing to their distinct features including low toxicity, inherent antibacterial, and angiogenic activity, drug loading with high capacity, as well as the potential for targeted drug delivery and controlled release of pharmaceutical agents via surface modification. Interestingly, some metal ions and elements such as Cu and Zn contribute to accelerating wound healing processes, when used as core and structural building blocks of MOFs [16,[291], [292], [293], [294], [295], [296], [297], [298], [299]].

### 7.1 Biocompatibility

Utilizing natural or synthetic, composite, or hybrid biomaterials as biocompatible scaffolds in tissue engineering and wound healing applications is currently limited by a number of factors, including weak cellular proliferation, poor differentiation, low mechanical stability and bioactivity. The development of nano-MOF-based scaffolds have recently shed light on the path of their commercialization, which made possible through addressing the aforementioned drawbacks owing to their distinct biocompatibility and distinguished physicomechanical and biochemical characteristics. Nano-MOFs could provide wound dressings with ion-releasing and drug-loading capacity, stem cell attachment, proliferation, and differentiation. They promote osteoconductivity, osteoinductivity, and wound therapeutic properties via enhancing antibacterial activity, drug loading capacity, and sustained drug release of the host scaffold [16,[300], [301], [302]].

Electrospun fibrous scaffolds are considered as highly appealing platforms for tissue regeneration, especially due to their relatively high aspect ratio and porosity [[303], [304], [305]]. Wang et al. [306] investigated the cytotoxicity of HKUST-1/chitosan/PVA fibrous mats by MTT assay using L929 fibroblast cells and proved their notable biocompatibility, cell adhesion, and proliferation. They also revealed an accelerated wound healing capability with low inflammatory response and an effective antibacterial activity against *S. aureus* and *E. coli*. In another research, Chen et al. [307] synthesized PCN-224(Zr/Ti)-loaded poly (lactic-co-glycolic acid) (PLGA) nanofibers by electrospinning method and examined its in-vivo biocompatibility through their tail intravenous injection to the lab rats. H&E staining of different organs including heart, liver, spleen, lung, and kidney indicated no signs of infection or severe inflammation. Furthermore, they evaluated biotoxicity of the nanoparticles using three biochemical parameters, including WBC (white blood cells), RBC (red blood cells), and PLT (platelets) and reported a normal range for all indices indicating a negligible biotoxicity. Cell viability tests on human umbilical vein endothelial cells (HUVECs) using commercial CCK-8 kit revealed promising biocompatibility of PCN-224(Zr/Ti) nanoparticles. Additionally, the hemolytic test revealed no evidence of damage on rat erythrocytes at the concentration of 320 μg/mL of the nanoparticles. The biocompatibility of a variety of other MOFs including ZIF-8 [308], ZIF-18, MIL-100 [309], MIL-88B [310], Mg-MOF-74 [311], PCN-222 [312], Uio-66(Zr) [313], nanoUiO-66-NH_2_ [314], and CAU-7 has also been investigated in the literature [315]. It is of note that the biocompatibility and biodegradability of MOFs for biomedical applications has been recently reviewed by Singh et al. [316].

### 7.2 Nano-MOFs for wound healing

**Fig. 8:**
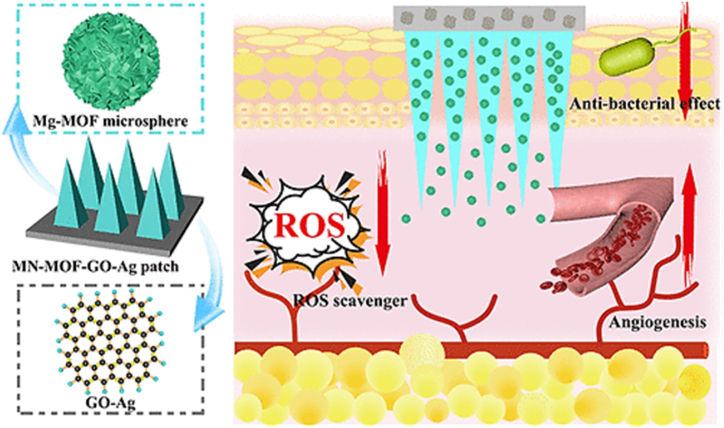
Schematic illustration of the wound healing mechanism for Mg-MOF-based microneedle patch (Reprinted with permission from Ref. [320]. Copyright 2021 American Chemical Society).

Because of their bactericidal and anti-inflammatory properties, MOF-based wound dressings promote cell proliferation, angiogenesis, and collagen deposition, all of which hasten wound healing [302,[317], [318], [319]]. To increase drug penetration depth to heal diabetic wound, Yin et al. [320] synthesized an Mg-MOF-based microneedle patch by loading Mg-MOFs containing poly(γ-glutamic acid) (γ-PGA) hydrogel into the tips and γ-PGA-GO-Ag nanocomposites into the backing layer, as shown in Fig. 8. Mg^2+^ ions and gallic acid were slowly released from the prepared microneedle patch into the dermis's deep layer, induced cell migration, and endothelial tubulogenesis, and promoted antioxidation. It should be noted that the incorporation of γ-PGA hydrogel and GO-Ag nanocomposite into the structure may contribute to considerably improved wound healing rate by providing outstanding antibacterial effects. In another study, Deng et al. [321] loaded AuNPs encapsulating MOFs (Au@ZIF-8) into an injectable alginate/gelatin hydrogel as an extracellular matrix for wound healing applications. As reported, the Au@ZIF-8 containing hydrogel promotes ROS release under visible light actuation compared with bare ZIF-8, which may be linked to its reinforced light absorption and charge carrier separation by Au-mediated surface plasmon resonance and Schottky junction. The synthesized composite demonstrated remarkable bactericidal activity against *E. coli* and *S. aureus* and significantly accelerated wound healing.

Free functional groups on MOFs' surface facilitate the surface engineering of MOFs through chemical and physical conjugation or adsorption of functional biological molecules. Surface-modified MOFs offer specific properties including targeted drug delivery, controlled release of pharmaceutical agents, additional angiogenic and antibacterial activity, lower toxicity, and higher drug loading capacity compared to other nanomaterials, making them appealing media for accelerated and cost-effective wound healing [299,[322], [323], [324], [325], [326]]. Prussian blue (PB)@MOFs core−shell structure [317], hyaluronic acid coating on Ag^+^ loaded MOFs [327], GRGDS-modified AgNPs-embedded γ-cyclodextrin (CD)-MOFs [328], niacin MOFs encapsulated microcapsules with alginate shells and Cu-/Zn-niacin framework cores [293], ZIF-8 loaded omniphobic porous hydrogel [298], Zn-MOFs encapsulated methacrylated hyaluronic acid microneedles array [294], porous GO-encapsulated copper-benzene-1,3,5-tricarboxylate MOF microneedle array patch [329], photosensitive hydrogels using MOFs modified with double-bond modified chitosan, Prussian blue, and quaternary ammonium [330], Cu-MOFs incorporated electrospun chitosan/PVA fibers [306], Cu-based NO-loaded electrospun core-shell MOFs surface modified with 4-(Methylamino) pyridine [331], drug (dimethyloxalylglycine, DMOG)-loaded ZIF-67 NPs containing micro-patterned PLLA/gelatin nanofibrous scaffolds [319], folic acid surface modified Cu-MOF NPs [332], Cu-MOFs/chitosan films [324], and metformin hydrochloride (MH) and curcumin incorporated Cu-MOFs impregnated into MH-loaded hydrogel [333] are among the novel formulations recently published in the field. MOFs’ applications in the healing process and their antibacterial functions in recently-developed wound dressings are represented in Table 8. In general, Mg/Cu-MOFs release Mg^2+^/Cu^2+^ ions through ion exchange in the wound environment. These ions play vital roles in lowering inflammation, promoting angiogenesis, preventing infection, and boosting collagen synthesis. The special structure of MOFs allows for controlled release and efficient absorption of wound fluid, making them promising materials for advanced therapeutic modalities [16,320,[334], [335], [336], [337], [338], [339]].

**Table 8:** Nanotechnology-driven advantages and antibacterial function of MOFs along with their effects on the accelerated wound healing mechanisms in advanced dressings.

Formulation	Microstructure properties	Nanotechnology-driven advantages	NPs antibacterial function	NPs function in accelerated wound healing mechanism	Ref.
Donut-like Cu-nicotinic acid (CuNA) MOF-bFGF@GelMA hydrogels	Metal ions: Cu ions; Average pore size: 200 μm	The incorporation of MOFs enhances the elastic properties of the hydrogels; The cell cytotoxicity and proliferation of GelMA hydrogels remain unaffected by the inclusion of MOFs and basic fibroblast growth factor (bFGF), while the migration, total segment length, and wound closure rate of HUVEC and NIH/3T3 cells increase.	CuNA@GelMAs hydrogels display effective antibacterial activity against *E. coli* and *S. aureus*; The antibacterial efficacy of the hydrogel improves with an increase in CuNA content.	The addition of MOFs and bFGF into GelMA promotes the formation of new blood vessels, regular epithelium, and mild inflammatory responses; The mechanisms involved include: (i) a reduction in the expression of interleukin-6 (IL-6) as a pro-inflammatory cytokine; (ii) an increase in Ki-67 expression as a cellular proliferation marker; and (iii) a stronger positive expression of CD31 and α-SMA, indicative of a more favorable angiogenesis response.	[340]
Electrospun MOF-chitosan/PVA nanofibrous membranes	Metal ions: Cu ions; Mean fiber diameter: 443 & 518 nm before and after incorporating MOFs, respectively.	The addition of MOF reduces the cell viability of chitosan/PVA fibers, attributed to increased Cu ions release. However, it accelerates the wound repair process by releasing Cu ions, which, in turn, promotes collagen deposition and stimulates angiogenesis.	Nanofibrous membranes display antibacterial activity against both *E. coli* and *S. aureus*; The incorporation of MOF enhances the antibacterial activity of the nanofibers.	–	[306]
PCN-224 (Zr/Ti) loaded PLGA nanofibers	–	Nanofibers containing MOF effectively hinder microbial invasion in infected wounds.	The nanofibers demonstrate antibacterial activity against a range of bacteria, including *E. coli*, *S. aureus*, S. epidermidis, A. baumannii, MRSA, MRSE (methicillin-resistant Staphylococcus epidermidis), and multiple drug-resistant *E. coli* & A. baumannii.	(i) ROS generation under visible light; (ii) reduction in inflammatory cytokines including TNF-α, IL-6 and IL-1β; and (iii) increase in lymphocytes and neutrophils during the healing process.	[307]

## 8 Clinical trials of nanoparticles in wound healing

Despite their valuable properties, the nanoparticles show deficits such as difficulty in monitoring and tuning the physicochemical properties, in-vivo behavior, toxicity, clinical scalability, commercialization capability, site-specificity, and reproducibility of such nanoscale systems. The appearance of cytotoxicity in the presence of excessive amounts of NPs in tissue environment is thought to be one of the most challenging of these drawbacks. To address these limitations, several formulations based on metallic-NPs including AgNPs, AuNPs, CuNPs, cerium oxide NPs, bioactive glass (BG), silicon (Si), and NO nanoparticles have been analyzed in-vivo using animal models for evaluating their pre-clinical efficacy in wound healing. However, there is no sufficient clinical data to prove their potential to be used unconditionally [341].

Among the above-discussed metallic nanoparticles, Ag-containing scaffolds have been at the center of attention and is considered as the only commercialized nanoparticles in wound dressing applications. In this regard, some clinical trials performed on evaluating the healing efficiency of the nanoparticles, according to the data recorded in FDA-supported ClinicalTrials.gov databank are summarized in Table 9. Noteworthy, many clinical studies are required to be conducted to provide sufficient evidence on the clinical effectiveness of these nanoparticles.

**Table 9:** Brief review of clinical trial studies on metallic-ions-releasing wound healing platforms recorded in FDA-supported ClinicalTrials.gov databank to date.

Nanomaterial	Study Title	Trial Phase	Recruitment status	Conditions	Interventions	Location
Silver	Comparative Study Using Negative Pressure Dressing With & Without Silver Alginate to Promote Healing in Chronic Wounds	Not Applicable^a^	Completed^b^	Wound Healing	Other: silver alginate; Other: VAC dressing without silver alginate	Dow University of Health Sciences, Pakistan
	A Comparative Efficacy and Safety Study Between Two Silver Containing Dressings in Post-Op Wound Healing	Not Applicable	Terminated^c^	Surgical Wound	Device: BCT Silver Bandage; Device: Aquacel® Ag Dressing	Chung-Shan Medical University Hospital Taichung City, Taiwan
	Evaluation of Diabetic Foot Wound Healing Using Hydrogel/Nano Silver-based Dressing vs. Traditional Dressing	Not Applicable	Completed	Diabetes Mellitus	Procedure: Hydrogel/nano silver-based dressing	Cairo University, Egypt
	An Open Single-center Investigation Evaluating Efficacy in Second Degree Partial Thickness Burns Using a Silicone Contact Layer Containing Silver.	Not Applicable	Completed	Wound Healing	Device: Mepilex Transfer Ag	Florida Gulf-to-bay Anesthesiology Tampa, US; Long Island Plastic Surgical Group, US
	A Comparison of Post-Sternotomy Dressings	Not Applicable	Completed	Impaired Wound Healing; Postoperative Wound Infection-deep	Other: Dry Sterile Dressing; Other: Metallic Silver Dressing; Other: Ionic Silver Dressing	Carilion Roanoke Memorial Hospital Roanoke, US
	Clinical and Radiographic Evaluation of the Synergistic Effect of Nano Silver Particles and Calcium Hydroxide Versus Triple Antibiotic Paste as Antibacterial Agents for Lesion Sterilization and Tissue Repair (LSTR) in Necrotic Primary Molars	Not Applicable	Not yet recruiting^d^	Root Canal Infection	Other: Nano silver particles and calcium hydroxide; Other: TAP	Cairo University, Egypt
	A Clinical Investigation to Evaluate Efficacy of Mepitel Ag in Partial Thickness Second Degree Burns	Not Applicable	Completed	Burns; Wound Healing	Device: Mepitel Ag	Arizona Burn Center Phoenix, US; Orlando Regional Medical Center, US; Wishard Indianapolis, US
	The Difference in Wound Size Reduction Comparing Two Frequently Used Wound Dressings in Everyday Care (wound-size)	Not Applicable	Completed	Wound Heal	Other: application of a polyacrylate wound pad; Other: Hydrocellular foam	Cité Génération Maison de santé Onex, Switzerland
Gold	31P-MRS Imaging to Assess the Effects of CNM-Au8 on Impaired Neuronal Redox State in Amyotrophic Lateral Sclerosis (REPAIR-ALS) (REPAIR-ALS)	Phase 2	Withdrawn^e^	Amyotrophic Lateral Sclerosis	Drug: Gold Nanocrystals	UT Southwestern Dallas, US
Copper & copper oxide	Efficacy of Wound Dressings with Copper Oxide	Not Applicable	Recruiting	Pressure Ulcer; Diabetic Foot Ulcer	Device: MedCu Antibacterial Wound Dressings with Copper Oxide	Loewenstein Rehabilitation Center Ra'anana, Israel
	Comparison of Wound Healing Between MedCu Dressings with Copper Oxide and Negative Pressure Wound Therapy Treatment	Not Applicable	Recruiting^f^	Wounds & Injuries; Negative Pressure Therapy; Diabetic Foot Ulcer	Device: MedCu wound dressings with copper oxide	Rambam Health Care Campus Haifa, Israel
Zinc & Zinc oxide	The Effect of Donepezil on Wound Healing	Phase 2	Recruiting	Wound Heal	Drug: Donepezil; Drug: Placebo oral tablet; Drug: Topical Zinc Oxide	University of Maryland Shore Medical Center at Easton, US
	Influence of Sun Protection and Linear Repair of Cutaneous Surgical Defects	Not Applicable	Recruiting	Surgical Wound; Wound Heal; Wound of Skin; Scar	Other: Zinc containing suncreen	University of California, Davis Sacramento, US
	Effect of EGF With Silver Sulfadiazine Cream Compared with Silver Zinc Sulfadiazine Cream for Treatment of Burn Wound	Phase 2; Phase 3	Completed	Deep Partial Thickness Burn	Drug: Epidermal growth factor with silver sulfadiazine cream; Drug: Silver zinc sulfadiazine cream	Burn Unit, Siriraj Hospital Bangkok, Thailand
	The Effects of Zn-containing Granulate on Patient Morbidity and Wound Healing After Free Gingival Graft Surgery	Not Applicable	Completed	Surgical Wound	Procedure: Zn granulate stent after FGG surgery; Procedure: Hemostatic agent suturing	Cukurova University Faculty of Dentistry Adana, Turkey

a Not Applicable is used to describe trials without FDA-defined phases, including trials of devices or behavioral interventions.

b The study has ended normally, and participants are no longer being examined or treated (that is, the last participant's last visit has occurred).

c The study has stopped early and will not start again. Participants are no longer being examined or treated.

d The study has not started recruiting participants.

e The study stopped early, before enrolling ts first participant.

f The study is currently recruiting participnts.

## 9 Limitations and side effects

Nanomaterials offer potential benefits for wound healing but also pose risks. Their cytotoxicity and allergenicity can hinder their application [342]. Studies have demonstrated that certain nanomaterials, such as AgNPs, can induce stress responses and be cytotoxic to mammalian cells specially at high concentrations through silver ions release [190]. Nanomaterials, such as SiO_2_NPs, TiO_2_NPs, and ZnONPs, have been implicated in skin inflammation, psoriasis, and hemolysis [[343], [344], [345]]. Additionally, nanomaterials may spread to unintended tissues, leading to adverse side effects including cytotoxicity or adverse immune responses, particularly at high concentrations or with prolonged exposure. These limitations highlight the need for careful evaluation and mitigation strategies before widespread clinical use of nanomaterials in wound healing [346].

While studies on nanomaterials for wound healing often focus on localized acute toxicity, evaluating systemic and chronic effects is equally important. Recent research highlights the need for comprehensive safety assessments, including long-term toxicity studies. This will enable a more informed understanding of the risks and benefits associated with nanomaterial-based wound treatments, guiding their safe and effective application. Factors such as nanomaterial type, usage patterns, and individual variations can influence the occurrence and severity of side effects. Therefore, careful consideration and consultation with healthcare professionals are essential when using nanomaterials in wound care [347].

While nanomaterials have demonstrated positive effects on wound healing in vitro, further in vivo studies are needed to fully understand their mechanisms of action and potential clinical applications. Animal models, such as rats and mice, have been commonly used, but their limitations in accurately representing human skin and wound healing processes should be considered. Standardizing wound models and investigating the role of nanomaterials in specific wound types, like diabetic wounds or immune skin diseases, will contribute to a deeper understanding of their therapeutic potential. Additionally, the mechanisms by which nanomaterials promote wound healing, beyond macrophage polarization and TGF-β1/SMAD signaling pathway, commonly reported pathways respectively in the inflammatory and proliferation phases, require further exploration. By elucidating these mechanisms, specially wound repair mechanism after reconstruction, researchers can develop more targeted and effective nanomaterial-based wound treatments [348].

## 10 Patents related to NPs application in wound healing

Nanomaterials-based antimicrobial wound dressings have great potential for future clinical use and an extremely large number of patents have been published to date (Table 10). The intellectual property landscape surrounding nanomaterials for wound healing is rapidly evolving. While significant progress has been made, there are still challenges to overcome before these technologies can be fully commercialized. Continued research and development, coupled with strategic intellectual property management, will be essential for realizing the full potential of nanomaterials in wound healing.

**Table 10:** Patents of NPs-based antimicrobial materials and wound dressings.

Patent Number	Title of Invention	Formulation	Nanotechnology-Driven Advantages	Publication Date
**US2024269342A1**	Green synthesis of Ag-Ni bimetallic nanoparticle-incorporated PVA/PAA hydrogels with antimicrobial properties	Ag-Ni bimetallic NPs incorporated in PVA/PAA hydrogel (NPs size: 12 nm)	Antibacterial properties; Effective against *E. coli*; Cooling effect for burns; Potential for promoting wound healing	2024-08-15
**CN115607687A**	Modified mesoporous silica silver-coated nano-particles loaded with siRNA and antibiotics as well as preparation method and application of modified mesoporous silica silver-coated nano-particles	PEG-g-PEI modified mesoporous silica silver-coated NPs loaded with siRNA and ciprofloxacin	Enhanced siRNA delivery and protection; Synergistic antibacterial effect of AgNPs and ciprofloxacin; Inhibition of TNF-alpha expression; Anti-inflammatory properties; Promoted wound healing; Low biotoxicity	2023-01-17
**CN111643720A**	Hydrogel with excellent antibacterial performance for burn wound healing and preparation method of hydrogel	(AgNPs & ZnNPs)-incorporated and Cyclosporine A (CsA)-loaded polyethylene glycol-N-isopropylacrylamide hydrogel	Excellent antibacterial performance; Promoted wound healing rate; Microbial resistance	2020-09-11
**RO133138A2**	Thermally active wound dressing based on silver nano-particles with ferromagnetic core, for treating skin lesions	Dressing containing Ag core-shell NPs Core: Nanocrystalline/amorphous ferromagnetic material	Thermally active wound dressing; Inductive heating by an electromagnetic field; Accelerated wound healing	2019-03-29
**US2016220606A1**	Silver-copper-zinc oxide wound care system	Ag, Cu, and Zn oxide colloidal NPs	Antimicrobial properties; Promotes wound healing; Maintains a moist environment	2016-08-04
**CN116570760B**	Multifunctional slow-release dressing for promoting chronic wound healing as well as preparation method and application of multifunctional slow-release dressing	Oxygen release hydrogel composed of PVA, sodium alginate, carboxymethyl chitosan, CaO_2_NPs, and TAX-coated MOF-199 NPs	Prominent oxygen release characteristics; Anti-inflammatory and antibacterial effects; Remarkably promotes wound healing; Potential for treating chronic wounds like diabetes mellitus	2023-08-11
**CN114272443B**	Preparation method and application of zinc silicate nanoparticle composite fiber scaffold	Zinc silicate NPs composite fiber scaffold	Promoted both skin wound healing and the regeneration of the skin nerve vascular network	2022-04-05
**US2023137084A1**	Nanoparticles to promote wound healing and antimicrobial infection control	Ag-modified CeO_2_NPs (NPs size: 3–35 nm)	Antimicrobial properties; Promoted wound healing; Combined with tissue adhesive or epithelial tissue healing agent	2023-05-04
**CN117959486A**	Epidermal growth factor/PDA-MXene temperature-sensitive hydrogel as well as preparation method and application thereof	Epidermal growth factor/PDA-MXene temperature-sensitive hydrogel based on poloxamer P407 and P188	Controlled release of epidermal growth factor; Temperature-sensitive gel for targeted drug delivery; NIR light-triggered release; Potential for treating chronic wounds	2024-05-03
**WO2022121255A1**	Chitosan/MXene antibacterial composite sponge for hemostasis, preparation method therefor and application thereof	Chitosan/MXene composite sponge	Antibacterial properties; Promoted hemostasis	2022-06-16
**CN115944772A**	Bacterial cellulose-polydopamine-MXene-coated AgNPs antibacterial hemostatic sponge as well as preparation method and application thereof	BC-PDA-MXene-AgNPs composite gel	Good mechanical properties and biocompatibility; Excellent antibacterial effect against *E. coli* and *S. aureus* under near-infrared light irradiation; Potential for treating skin wounds with bacterial infections	2023-04-11
**CN111925557B**	Preparation method of silk fibroin wound dressing based on MXene enhancement	Silk fibroin/MXene composite fiber scaffold	Improved mechanical and electrical properties; Enhanced biocompatibility; Antibacterial and anti-inflammatory effects; Potential for tissue engineering applications	2020-11-13
**CN118079075A**	Nano-reactor hydrogel for promoting healing of diabetic wounds as well as preparation method and application of nano-reactor hydrogel	(Arginine & glucose oxidase)-incorporated Zn-MOF injectable nano-reactor hydrogel	Responsive to hyperglycemia; Reduces blood sugar and pH; Generates NO for anti-inflammatory and angiogenesis effects; Good biocompatibility and bacterial proliferation resistance; Can be used for visual monitoring of wound healing	2024-05-28
**CN112250887B**	Copper metal organic framework nanoparticle functionalized hydrogel, preparation method and application thereof	(Cu-MOF-NPs)-incorporated carboxymethyl chitosan-based hydrogel matrix surface modified with glutathione	Controlled release of Cu ions; Removal of active oxygen from wound surface; Maintenance of a moist 3D microenvironment; Protection of the wound from external injury; Promotion of wound healing	2021-01-22

## 11 Future directions and commercialization perspectives

By enhancing platelet aggregation and activating clotting factors, nanomaterials can accelerate hemostasis. During the inflammatory phase, they can regulate inflammation, inhibit bacterial growth, and promote wound cleansing. In the proliferation phase, nanomaterials provide a supportive environment for cell growth and tissue regeneration. Finally, in the remodeling phase, they can modulate cell differentiation and extracellular matrix reconstruction, facilitating the development of functional tissue. These findings highlight the potential of nanomaterials as innovative therapeutic agents for wound healing.

While nanomaterials offer promising potential for wound healing, several challenges hinder their clinical translation. These challenges include manufacturing costs, stability, biocompatibility, toxicity, and regulatory hurdles. Addressing these issues through advancements in characterization techniques, formulation optimization, and rigorous testing is crucial for advancing the clinical application of nanomaterials in wound care.

Obtaining regulatory approval for nanomaterial-based wound dressings can be a complex and time-consuming process due to safety and efficacy concerns. Furthermore, the production of nanomaterials can be expensive, potentially limiting their widespread adoption in healthcare settings. Ensuring consistent quality and reproducibility of nanomaterial-based wound dressings can be also challenging due to variations in synthesis methods and material properties.

While nanomaterials show promise in wound healing, their use during pregnancy requires further investigation due to potential placental crossing and fetal development concerns. Animal models, while informative, may not fully represent human wound healing dynamics, especially in elderly or immunocompromised patients. Additionally, the interaction between nanomaterials and other medications used in wound care needs to be explored. To fully understand the safety and efficacy of nanomaterials in wound healing, more clinical studies and human trials are essential.

To provide an understanding of the economic factors associated with nanomaterial-based wound dressings, the factors influencing their pricing should be addressed. The factors include.

i. Material composition: The type and purity of the nanomaterials used can significantly impact production costs.
ii. Synthesis method: The complexity and efficiency of the synthesis process can influence the overall cost.
iii. Production scale: Larger-scale production can often lead to economies of scale and lower unit costs.
iv. Intellectual property: Patents and other intellectual property rights associated with nanomaterial production may contribute to higher costs.
v. Regulatory requirements: Compliance with regulatory standards can add to the overall cost of nanomaterial production.

By considering these factors, researchers could offer insights into the potential for cost reduction and the economic implications of nanomaterial-based wound healing solutions.

## 12 Conclusions

There has always been a growing enthusiasm among chemical, materials, and biological engineers to design novel nano-systems based on new compositions and formulations and subsequently develop innovative material platforms for a wide range of existing applications, especially tissue engineering and wound healing. Due to the noticeable characteristics of nanomaterials, various types of nano-engineered wound dressings have been contrived based on emerging NPs, including M/MO-NPs, MXene nanosheets, and MOFs.

The incorporation of various types of nanoparticles into wound healing biomaterials has demonstrated significant potential in affecting key aspects of the healing process. Metallic nanoparticles, renowned for their antimicrobial properties, significantly contribute to lowered infection risk at wound sites. Concurrently, metal oxide NPs play a pivotal role in regulation of inflammation and facilitation of tissue regeneration, presenting a multifaceted approach for enhancing the healing cascade. MXene nanoparticles have emerged as a noteworthy addition to wound care biomaterials, showcasing the ability to enhance cell adhesion and proliferation. The unique physicochemical properties of MXene contribute to improved cellular interactions, fostering an environment conducive to accelerated tissue repair. Moreover, MOFs exhibit promise in the realm of controlled drug delivery, providing a platform for precise and sustained release of therapeutic agents. This capability enhances the therapeutic potential of wound dressings, allowing for targeted treatment and prolonged efficacy.

While the literature suggests the possible advantages of incorporating these diverse nanoparticles into wound healing applications, it is imperative to acknowledge the need for further research. A comprehensive understanding of the underlying mechanisms governing the actions of metallic, metal oxide, MXene, and MOF nanoparticles is of paramount importance. Additionally, optimizing the utilization of these nanoparticles in composite biomaterials requires a nuanced exploration of formulation parameters, concentrations, and synergistic effects to maximize their therapeutic impact.

In conclusion, the current state of research underscores the promise of metallic, metal oxide, MXene, and MOF nanoparticles in advancing wound healing strategies. Notwithstanding, to fully harness their potential, ongoing investigations are warranted to unravel the intricate mechanisms at play and refine the design principles guiding their integration into biomaterials tailored for effective wound management.

## CRediT authorship contribution statement

**Gholamreza Faghani:** Writing – review & editing, Supervision. **Amir Azarniya:** Writing – original draft.

## Data availability statement

The data used to support the findings of this study are included within the article.

## Declaration of competing interest

The authors declare that they have no known competing financial interests or personal relationships that could have appeared to influence the work reported in this paper.
